# Safety, Tolerability, and Pharmacokinetics of Senaparib, a Novel PARP1/2 Inhibitor, in Chinese Patients With Advanced Solid Tumors: A Phase I Trial

**DOI:** 10.1093/oncolo/oyad163

**Published:** 2023-06-20

**Authors:** Junning Cao, Hongqian Guo, Dongmei Ji, Weina Shen, Shun Zhang, Chih-Yi Hsieh, Sui Xiong Cai, Ye Edward Tian, Cong Xu, Pin Zhang, Binghe Xu

**Affiliations:** Department of Medical Oncology, Fudan University Shanghai Cancer Center Shanghai, Shanghai, People’s Republic of China; Department of Urology, Nanjing Drum Tower Hospital, Clinical College of Nanjing Medical University, Nanjing, People’s Republic of China; Department of Medical Oncology, Fudan University Shanghai Cancer Center Shanghai, Shanghai, People’s Republic of China; Department of Medical Oncology, Fudan University Shanghai Cancer Center Shanghai, Shanghai, People’s Republic of China; Department of Urology, Nanjing Drum Tower Hospital, Clinical College of Nanjing Medical University, Nanjing, People’s Republic of China; IMPACT Therapeutics Inc., Shanghai, People’s Republic of China; IMPACT Therapeutics Inc., Shanghai, People’s Republic of China; IMPACT Therapeutics Inc., Shanghai, People’s Republic of China; IMPACT Therapeutics Inc., Shanghai, People’s Republic of China; Department of Medical Oncology, National Cancer Center/National Clinical Research Center for Cancer/Cancer Hospital, Chinese Academy of Medical Sciences and Peking Union Medical College, Beijing, People’s Republic of China; Department of Medical Oncology, National Cancer Center/National Clinical Research Center for Cancer/Cancer Hospital, Chinese Academy of Medical Sciences and Peking Union Medical College, Beijing, People’s Republic of China

**Keywords:** PARP inhibitor, senaparib, solid tumors, *BRCA* mutation, synthetic lethality, phase I study

## Abstract

**Background:**

Senaparib, a novel poly(ADP-ribose) polymerase 1/2 inhibitor, demonstrated antitumor activity in preclinical studies. This phase I, first-in-human, dose-escalation/-expansion study explored the pharmacokinetics, safety and tolerability, and preliminary antitumor activity of senaparib in Chinese patients with advanced solid tumors.

**Patients and Methods:**

Adults with advanced solid tumors who had failed ³1 line of prior systemic treatment were enrolled. Senaparib (once daily [QD]) dose was escalated from 2 mg until the maximum tolerated dose (MTD)/recommended phase II dose (RP2D) using a modified 3 + 3 design. Dose expansion included: dose groups with ≥1 objective response and one dose higher, as well as those at the MTD/RP2D. Primary objectives were to evaluate the safety and tolerability, and determine the MTD and/or RP2D of senaparib.

**Results:**

Fifty-seven patients were enrolled across 10 dose groups (2-120 mg QD, and 50 mg twice daily). No dose-limiting toxicities were observed. The most common senaparib-related adverse events were anemia (80.9%), white blood cell count decreased (43.9%), platelet count decreased (28.1%), and asthenia (26.3%). Senaparib exposure increased dose proportionately at 2-80 mg; absorption saturated at 80-120 mg. Senaparib accumulation was minimal after repeated QD administration (accumulation ratio=1.1-1.5). The objective response rate was 22.7% (*n*=10/44) overall (all partial responses) and 26.9% (*n*=7/26) for patients harboring *BRCA1*/*BRCA2* mutations. Disease control rates were 63.6% and 73.1%, respectively.

**Conclusions:**

Senaparib was well tolerated and demonstrated promising antitumor activity in Chinese patients with advanced solid tumors. The RP2D for this clinical study in China was identified as 100 mg QD.

**ClinicalTrials.gov Identifier:**

NCT03508011

**Implications for Practice**

This first-in-human study demonstrated that senaparib was well tolerated in patients with advanced solid tumors. Moreover, there were encouraging signs of antitumor efficacy and disease control; 10 of the 44 evaluable patients achieved a partial response and another 18 had stable disease. Patients harboring *BRCA1*/*BRCA2* mutations appeared to receive particular benefits, as well as those with ovarian compared with non-ovarian tumors. These preliminary findings support further evaluation of this novel poly(ADP ribose) polymerase 1/2 inhibitor in larger populations with advanced solid tumors, and potentially in populations enriched for patients with *BRCA1*/*BRCA2* mutations and/or ovarian cancer.

## Introduction

Synthetic lethality is an important concept in precision oncology that is exploited to selectively kill cancer cells. It refers to a genetic interaction whereby loss or inactivation of either of 2 genes does not impact cell viability, but simultaneous loss or inactivation of both leads to cell death.^1,2^ One example of a synthetically lethal interaction is that between poly(ADP-ribose) polymerase (PARP) and the breast cancer susceptibility genes *BRCA1* and *BRCA2* (BRCA), which are involved in the repair of DNA single-strand and double-strand breaks, respectively.^3,4^ Inactivation of one of these DNA-repair pathways renders cells dependent upon the other pathway, and simultaneous inactivation results in genetic instability and promotes apoptosis. Therefore, in cells with BRCA mutations (BRCA^mut+^), PARP inhibition can lead to cell death.^4^

A variety of solid tumors harbor alterations in BRCA genes.^5-7^ In a recent pan-cancer analysis (*n* = 234; 154 specimens from 408 tumor types), the overall proportion with BRCA^mut+^ was 4.7%, the highest incidence being found for ovarian (15.2%), prostate (10.7%), skin squamous cell (9.1%), breast (8.8%), endometrial (5.3%), and pancreatic (5.2%) cancers.^8^ In some indications, including ovarian, breast, and prostate cancers, these alterations have been shown to confer sensitivity to PARP inhibition.^9-14^

Senaparib (formerly IMP4297) is a novel oral PARP(1/2) inhibitor that demonstrated strong antitumor activity in preclinical studies, and particularly in BRCA^mut+^ tumors. Preclinical pharmacologic studies showed that senaparib was 20-fold more potent than the PARP inhibitor olaparib in in vivo studies (anticancer animal models), and that it is highly selective, and thus has a low probability of causing off-target-related adverse events.^15^ A phase I, first-in-human study was conducted to evaluate the safety and tolerability, pharmacokinetic (PK) profile, and antitumor activity of senaparib in Chinese patients with advanced solid tumors.

## Methods

### Study Design and Patients

This phase I, open-label, multicenter, dose-escalation, and dose-expansion study was conducted at three national clinical institutions in China. Patients aged 18-75 years with histologically or cytologically confirmed advanced solid tumors who had failed at least one line of prior systemic treatment were enrolled. At baseline, all patients enrolled in the dose-escalation phase had to have at least one evaluable lesion, and those in the dose-expansion period had to have at least one measurable lesion, according to Response Evaluation Criteria in Solid Tumors version 1.1 (RECIST v1.1).^16^ Entry to the dose-expansion cohort required the presence of a BRCA mutation, with the exception of patients with ovarian carcinoma, fallopian tube carcinoma, and primary peritoneal carcinoma. Full study eligibility criteria are provided in Supplementary Table S1.

Dose escalation was conducted using a modified Fibonacci 3 + 3 method (Supplementary Fig. S1).^17^ Patients were administered oral senaparib from a planned starting dose of 2 mg once daily (QD), with preliminary dose escalation to 120 mg via nine dose groups, in order of their entry into the study. At a given dose level, each patient received a single dose of senaparib followed by a 7-day washout period. They then entered a continuous dosing period to confirm the safety and PK profile, in which they received senaparib QD from day 1 to day 21 of each 3-week cycle. Progression to the next higher dose required approval from the Data Safety Monitoring Committee (DSMC) upon review of the safety and PK data of the previous dose. A 50-mg twice-daily (BID) dosing group was added (planned enrollment, *n* = 6 patients) based on a DSMC review of the safety, PK, and efficacy analysis of the completed QD dose groups in the phase I clinical trial of senaparib dose escalation in Australia (NCT03507543), which commenced prior to the start of this trial.

The dose-expansion period included 3 populations: dose groups in which at least one patient achieved complete response (CR) or partial response (PR) per RECIST v1.1^16^ and 1 dose group higher (evaluated for safety), as well as the dose group at the maximum tolerated dose (MTD)/recommended phase II dose (RP2D). Each of these groups included those patients in the respective dose-escalation group plus a planned additional 10 patients (approximately 20 patients in the MTD/RP2D group) with BRCA^mut+^ solid tumors (ovarian, breast, or prostate cancer were preferred, and ovarian cancer could be BRCA wild type). Patients received senaparib monotherapy orally QD or BID (50-mg dose only) over each 3-week cycle until disease progression or unacceptable toxicity.

This trial was conducted in accordance with the ethical principles for medical research involving human subjects stated in the Declaration of Helsinki, the ethical requirements of the Chinese Good Clinical Practice guidelines, and the Technical Guidelines for Clinical Trials of Antineoplastic Drugs issued by the National Medical Products Administration. All patients provided written informed consent to participate. The trial is registered with ClinicalTrials.gov (NCT03508011).

### Study Objectives and Assessments

The primary objectives of this study were to evaluate the safety and tolerability of senaparib in patients with advanced solid tumors, and to determine the MTD and/or the RP2D for clinical trials in China. The PK profile of senaparib after single and multiple doses, and preliminary antitumor efficacy was also assessed.

Safety was assessed by recording the incidence of dose-limiting toxicities (DLTs), and the incidence, nature, severity (graded per National Cancer Institute Common Terminology Criteria for Adverse Events version 4.03 [NCI CTCAE v4.03]^18^), and relatedness of treatment-emergent adverse events (TEAEs). DLTs were predefined toxicities (Supplementary Table S2) recorded over the 7 days following the single-dose administration of senaparib and during the first treatment cycle (day 1-day 21) for each dose group in the dose-escalation period only. The MTD was defined as the maximum dose at which ≤1/6 patients experienced a DLT. Senaparib PK parameters were derived from blood samples collected from patients after the single administration, and at regular intervals during the first cycle of the dose-escalation and dose-expansion periods. Plasma concentrations of senaparib were determined using high-performance liquid chromatography-tandem mass spectrometry. Tumor response was assessed every 6 weeks per RECIST v1.1,^16^ recording objective response rate (ORR), disease control rate (DCR), duration of response (DOR), and progression-free survival (PFS) by dose level for all patients and for those harboring BRCA^mut+^ tumors.

The RP2D was determined based on all efficacy, safety, and PK data.

### Statistical Analysis

There was no formal sample size calculation based on statistical hypothesis testing for this study; the planned enrolment was approximately 30-60 patients across the dose-escalation and dose-expansion cohorts. The actual number in the dose-escalation cohort was adjusted based on the number of dose cohorts required for the modified Fibonacci dose-escalation design and the determined MTD. Definitions for all analysis populations (safety, DLT, PK, and efficacy [intent-to-treat, ITT]) are provided in Supplementary Table S2. No statistical tests were performed; patient demographic and baseline characteristics, safety/tolerability, PK parameters, and efficacy were evaluated descriptively. ORR and DCR and their 95% CIs were calculated for each group using the exact probability method. Kaplan-Meier statistics were used to estimate DOR and PFS, and their 95% CIs for each dose group separately. Dose linearity was explored based on the single-dose data of area under the time-concentration curve (AUC) and maximum plasma concentration (*C*_max_) using the power model (*Y* = *α* × dose *β*) for the dose range 2-80 mg on a log-transformed scale. The following criterion was used to determine whether dose proportionality was met: if the (2-sided) 90% CI for *β* was wholly contained within the interval (1 + log(0.5)/log(*r*), 1 + log(2)/log(*r*)), then dose proportionality was supported. Here, *r* was defined as the ratio of the highest to lowest dose. All data analyses were carried out using SAS EG7.1 or higher or (for PK parameters) WinNonlin 7.0 or higher.

## Results

### Patients and Treatment

**Figure 1.**
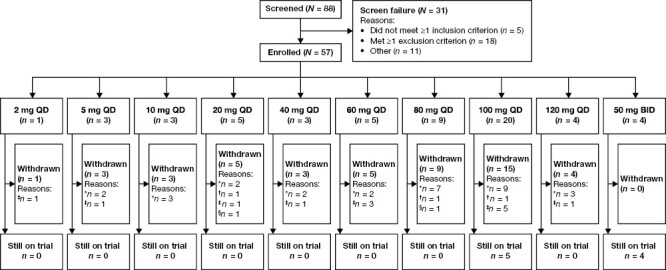
Patient disposition. *Evidence of disease progression. ^**†**^Intolerable toxicity or cumulative toxicity preventing the patient from continuing. ^**‡**^Patient or physician decision to discontinue for other reasons. ^**§**^Patient death. BID, twice daily; QD, once daily.

Between August 30, 2017 and June 9, 2020, 57 of the 88 screened patients were enrolled (33 patients in the dose-escalation period and 24 in the dose-expansion period) (Fig. 1). The 20-, 60-, 80-, and 100-mg QD dose groups were evaluated in the dose-expansion period. The most frequent primary cancers were ovarian (*n* = 28, 49.1%) and breast (*n* = 16, 28.1%), and the majority of patients had stage IV disease (*n* = 56, 98.2%). All patients had metastases at baseline, BRCA^mut+^ was confirmed in 32 (56.1%) patients, and 35 (61.4%) had received at least three prior lines of systemic anticancer therapy (Table 1).

**Table 1.** Demographic and baseline characteristics of all patients (dose-escalation and dose-expansion periods)

Characteristic	Dose group										
	2 mg QD *n* = 1	5 mg QD *n* = 3	10 mg QD *n* = 3	20 mg QD *n* = 5	40 mg QD *n* = 3	60 mg QD *n* = 5	80 mg QD *n* = 9	100 mg QD *n* = 20	120 mg QD *n* = 4	50 mg BID^a^ *n* = 4	Total *N* = 57
Age (years) median (Q1, Q3)	60.0 (60.0, 60.0)	62.0 (50.0, 68.0)	58.0 (58.0, 59.0)	55.0 (48.0, 62.0)	46.0 (45.0, 59.0)	49.0 (46.0, 49.0)	47.0 (46.0, 60.0)	53.5 (49.0, 61.5)	53.0 (42.0, 62.5)	63.5 (56.0, 67.5)	54.0 (47.0, 61.0)
Sex, female *n* (%)	1 (100)	1 (33.3)	2 (66.7)	5 (100)	2 (66.7)	5 (100)	8 (88.9)	19 (95.0)	4 (100)	1 (25.0)	48 (84.2)
Ethnicity, *n* (%)											
Han	1 (100)	3 (100)	3 (100)	4 (80.0)	3 (100)	5 (100)	8 (88.9)	20 (100)	4 (100)	4 (100)	55 (96.5)
Other	0	0	0	1 (20.0)	0	0	1 (11.1)	0	0	0	2 (3.5)
ECOG PS											
0	0	0	0	1 (20.0)	2 (66.7)	0	4 (44.4)	6 (30.0)	3 (75.0)	0	16 (28.1)
1	1 (100)	3 (100)	3 (100)	4 (80.0)	1 (33.3)	5 (100)	5 (55.6)	14 (70.0)	1 (25.0)	4 (100)	41 (71.9)
Primary tumor											
Ovarian	1 (100)	1 (33.3)	1 (33.3)	2 (40.0)	0	3 (60.0)	6 (66.7)	13 (65.0)	1 (25.0)	0	28 (49.1)
Breast	0	0	1 (33.3)	3 (60.0)	2 (66.7)	1 (20.0)	1 (11.1)	5 (25.0)	2 (50.0)	1 (25.0)	16 (28.1)
Prostate	0	2 (66.7)	1 (33.3)	0	1 (33.3)	0	0	1 (5.0)	0	3 (75.0)	8 (14.0)
Other^b^	0	0	0	0	0	1 (20.0)	2 (22.2)	1 (5.0)	1 (25.0)	0	5 (8.8)
Overall tumor staging											
IV	1 (100)	3 (100)	3 (100)	4 (80.0)	3 (100)	5 (100)	9 (100)	20 (100)	4 (100)	4 (100)	56 (98.2)
IIIc	0	0	0	1 (20.0)	0	0	0	0	0	0	1 (1.8)
Metastasis at baseline											
Yes, *n* (%)	1 (100)	3 (100)	3 (100)	5 (100)	3 (100)	5 (100)	9 (100)	20 (100)	4 (100)	4 (100)	57 (100)
Prior lines of therapy											
1-2	0	1 (33.3)	1 (33.3)	2 (40.0)	0	3 (60.0)	4 (44.4)	6 (30.0)	3 (75.0)	2 (50.0)	22 (38.6)
≥3	1 (100)	2 (66.7)	2 (66.7)	3 (60.0)	3 (100)	2 (40.0)	5 (55.6)	14 (70.0)	1 (25.0)	2 (50.0)	35 (61.4)
BRCA mutation status^c^											
Positive	0	2 (66.7)	1 (33.3)	3 (60.0)	0	4 (80.0)	6 (66.7)	13 (65.0)	2 (50.0)	1 (25.0)	32 (56.1)
Negative	1 (100)	1 (33.3)	2 (66.7)	2 (40.0)	3 (100)	1 (20.0)	3 (33.3)	7 (35.0)	2 (50.0)	3 (75.0)	25 (43.9)

^a^Dose group added as a result of analysis of safety, PK, and efficacy of the completed QD dose groups in the dose-escalation period of the phase I Australian trial (NCT03507543).

^b^Includes two patients with cervical cancer (60-mg and 80-mg dose groups), and one each with bladder cancer (80-mg dose group), lung cancer (100-mg dose group), and vaginal cancer (120-mg dose group).

^c^BRCA mutation status of blood samples collected at baseline and tested by previous local testing or at a central laboratory.

Abbreviations: BID, twice daily; BRCA, *BRCA1* and/or *BRCA2*; ECOG PS, Eastern Cooperative Oncology Group performance status; PK, pharmacokinetic; Q1, Q3, interquartile range; QD, once daily.

As of June 9, 2020, nine (15.8%) patients were still on trial (4 in the 50-mg BID group in the dose-escalation period and 5 in the 100-mg group in the dose-expansion period) (Fig. 1). All 57 patients were included in the safety, PK, and ITT analysis sets; the DLT analysis set included all 33 patients enrolled in the dose-escalation period.

### Safety

The mean ± SD duration of exposure to senaparib was 126.5 ± 122.2 days (range, 22-576 days) across the dose-escalation and dose-expansion periods (*N* = 57; Supplementary Table S3). No DLTs were observed during the DLT observation period of the study. The MTD was not reached. As senaparib exposure appeared to plateau at 80-120 mg (based on PK data), the dose was not escalated further. Antitumor activity was observed at doses of 20 mg QD and above. The RP2D of senaparib was determined to be 100 mg QD.

All 57 patients enrolled in the study experienced at least one TEAE (*n* = 491 events) (Supplementary Table S4). The most common TEAEs were anemia (*n* = 29, 80.9%), white blood cell count (WBC) decreased (*n* = 25, 43.9%), platelet count decreased (*n* = 17, 29.8%), and asthenia (*n* = 16, 28.1%). Grade ≥3 TEAEs were reported in 27 (47.4%) patients, most frequently anemia (*n* = 12, 21.1%), neutrophil count decreased (*n* = 3, 5.3%), and platelet count decreased (*n* = 3, 5.3%). All other grades ≥3 TEAEs occurred in ≤2 (3.5%) patients each (Supplementary Table S5). The majority of TEAEs had resolved or stabilized at the time of data collection.

Fifty-five (96.5%) patients experienced senaparib-related adverse events (TRAEs) (Table 2). The most common TRAEs were anemia (*n* = 29, 80.9%), WBC decreased (*n* = 25, 43.9%), and platelet count decreased (*n* = 16, 28.1%). The overall incidence of grade ≥3 TRAEs was 33.3% (*n* = 19); the most frequently reported was anemia (*n* = 12, 21.1%). There were no grade ≥3 TRAEs in the 2-, 10-, 40-, and 60-mg dose groups, and the highest incidence was observed in the 120-mg dose group (*n* = 3, 75.0%). Serious adverse events were experienced by 12 (21.1%) patients and were considered to be related to senaparib in seven (12.3%).

**Table 2.** Incidence of senaparib-related treatment-emergent adverse events occurring in ≥10% of patients, any grade and grade ≥3, by preferred term (dose escalation and dose expansion phases; safety analysis set, *N* = 57)

TRAE (by preferred term), *n* (%)	Senaparib dose group																					Total *N* = 57
	2 mg QD *n* = 1		5 mg QD *n* = 3			10 mg QD *n* = 3		20 mg QD *n* = 5		40 mg QD *n* = 3		60 mg QD *n* = 5		80 mg QD *n* = 9		100 mg QD *n* = 20		120 mg QD *n* = 4		50 mg BID *n* = 4		
	All	Gr≥3	All	Gr≥3	All	Gr≥3	All	Gr≥3	All	Gr≥3	All	Gr≥3	All	Gr≥3	All	Gr≥3	All	Gr≥3	All	Gr≥3	All	Gr≥3
Any	**1** **(100)**	**0**	**3** **(100)**	**1** **(33.3)**	**3** **(100)**	**0**	**3** **(60.0)**	**1** **(20.0)**	**3** **(100)**	**0**	**5** **(100)**	**0**	**9** **(100)**	**5** **(55.6)**	**20** **(100)**	**7** **(35.0)**	**4** **(100)**	**3** **(75.0)**	**4** **(100)**	**2** **(50.0)**	**55** **(96.5)**	**19** **(33.3)**
Anemia^a^	**0**	**0**	**2** **(66.7)**	**1** **(33.3)**	**1** **(33.3)**	**0**	**1** **(20.0)**	**1** **(20.0)**	**1** **(33.3)**	**0**	**0**	**0**	**7** **(77.8)**	**3** **(33.3)**	**11** **(55.0)**	**4** **(20.0)**	**3** **(75.0)**	**1** **(25.0)**	**3** **(75.0)**	**2** **(50.0)**	**29** **(80.9)**	**12** **(21.1)**
WBC decreased	**0**	**0**	**1** **(33.3)**	**0**	**1** **(33.3)**	**0**	**1** **(20.0)**	**0**	**0**	**0**	**2** **(40.0)**	**0**	**5** **(55.6)**	**1** **(11.1)**	**9** **(45.0)**	**1** **(5.0)**	**4** **(100)**	**0**	**2** **(50.0)**	**0**	**25** **(43.9)**	**2** **(3.5)**
Platelet count decreased	**0**	**0**	**1** **(33.3)**	**0**	**0**	**0**	**1** **(20.0)**	**0**	**0**	**0**	**2** **(40.0)**	**0**	**4** **(44.4)**	**2** **(22.2)**	**6** **(30.0)**	**1** **(5.0)**	**1** **(25.0)**	**0**	**1** **(25.0)**	**0**	**16** **(28.1)**	**3** **(5.3)**
Asthenia	**0**	**0**	**1** **(33.3)**	**0**	**0**	**0**	**1** **(20.0)**	**0**	**1** **(33.3)**	**0**	**1** **(20.0)**	**0**	**2** **(22.2)**	**0**	**5** **(25.0)**	**0**	**2** **(50.0)**	**0**	**1** **(25.0)**	**0**	**15** **(26.3)**	**0**
Nausea	**0**	**0**	**1** **(33.3)**	**0**	**0**	**0**	**1** **(20.0)**	**0**	**0**	**0**	**1** **(20.0)**	**0**	**4** **(44.4)**	**0**	**4** **(20.0)**	**0**	**1** **(25.0)**	**0**	**2** **(50.0)**	**0**	**14** **(24.6)**	**0**
Appetite decreased	**1** **(100)**	**0**	**1** **(33.3)**	**0**	**1** **(33.3)**	**0**	**0**	**0**	**0**	**0**	**1** **(20.0)**	**0**	**3** **(33.3)**	**0**	**5** **(25.0)**	**0**	**1** **(25.0)**	**0**	**0**	**0**	**13** **(22.8)**	**0**
Neutrophil count decreased	**0**	**0**	**1** **(33.3)**	**0**	**0**	**0**	**0**	**0**	**0**	**0**	**1** **(20.0)**	**0**	**4** **(44.4)**	**1** **(11.1)**	**5** **(25.0)**	**2** **(10.0)**	**1** **(25.0)**	**0**	**0**	**0**	**12** **(21.1)**	**3** **(5.3)**
ALT increased	**0**	**0**	**0**	**0**	**1 (33.3)**	**0**	**0**	**0**	**0**	**0**	**0**	**0**	**1 (11.1)**	**0**	**5 (25.0)**	**0**	**2 (50.0)**	**1 (25.0)**	**1 (25.0)**	**0**	**10 (17.5)**	**1 (1.8)**
Vomiting	**0**	**0**	**0**	**0**	**0**	**0**	**0**	**0**	**0**	**0**	**0**	**0**	**2 (22.2)**	**1 (11.1)**	**5** **(25.0)**	**0**	**1** **(25.0)**	**0**	**2** **(50.0)**	**0**	**10** **(17.5)**	**1** **(1.8)**
AST increased	**0**	**0**	**0**	**0**	**1** **(33.3)**	**0**	**0**	**0**	**0**	**0**	**0**	**0**	**2** **(22.2)**	**0**	**4** **(20.0)**	**1** **(5.0)**	**2** **(50.0)**	**0**	**1** **(25.0)**	**0**	**10** **(17.5)**	**1** **(1.8)**
Blood creatinine increased	**0**	**0**	**0**	**0**	**0**	**0**	**1** **(20.0)**	**0**	**1** **(33.3)**	**0**	**1** **(20.0)**	**0**	**2** **(22.2)**	**0**	**2** **(10.0)**	**0**	**1** **(25.0)**	**0**	**0**	**0**	**8** **(14.0)**	**0**
Blood bilirubin increased	**0**	**0**	**1** **(33.3)**	**0**	**0**	**0**	**0**	**0**	**0**	**0**	**0**	**0**	**2** **(22.2)**	**0**	**3** **(15.0)**	**0**	**2** **(50.0)**	**1** **(25.0)**	**0**	**0**	**8** **(14.0)**	**1** **(1.8)**
Hypokalemia	**1** **(100)**	**0**	**0**	**0**	**0**	**0**	**1** **(20.0)**	**0**	**0**	**0**	**1** **(20.0)**	**0**	**3** **(33.3)**		**0**	**0**	**0**	**0**	**0**	**0**	**6** **(10.5)**	**0**

^a^Includes both anemia and hemoglobin decreased.

Abbreviations: ALT, alanine aminotransferase; AST, aspartate aminotransferase; BID, twice daily; Gr≥3, grade ≥3; QD, once daily; TRAE, treatment (senaparib)-related adverse event; WBC, white blood cell.

TEAEs causing dose interruption, dose reduction, and study drug discontinuation occurred in 15 (26.3%), 8 (14.0%), and 6 (10.5%) patients, respectively. The only TEAE causing study drug discontinuation in more than one patient across the dose groups was anemia (*n* = 3, 5.3%; 1 patient in the 20-mg group, 1 in the 80-mg group, and 1 in the 100-mg group).

TEAEs resulted in death for 2 (3.5%) patients, 1 in the 20-mg dose group (advanced disease/hemorrhagic shock), and 1 in the 80-mg dose group (disease progression/ileus—unconfirmed). Neither was considered to be related to senaparib.

### PK Properties

Details of PK parameters for the single-dose period are provided in Supplementary Table S6. Median time to *C*_max_ following a single dose of 2-120 mg (including 50 mg BID) ranged between 0.5 and 6.0 h. Senaparib exposure, as reflected by *C*_max_ and AUC, tended to increase over the dose range of 2-80 mg, and appeared to plateau, indicating saturation, at 80-120 mg (Supplementary Fig. S2; and Table S6). The elimination half-life ranged from 5.1 to 24.3 h, and mean apparent clearance and volume of distribution were 1.0-6.3 L/h and 15.5-88.7 L, respectively (dose range 2-120 mg).

Details of PK parameters for the continuous dosing period are provided in Supplementary Table S7. In this period, the exposure profile in the steady-state phase (day 15) was similar to that on day 1 (Supplementary Fig. S3). AUC from time zero to 24 h post dose in the 50-mg BID group was approximately 35 918 h*ng/mL, which was similar to the AUC from time zero to the last measurable concentration of 33 827 h*ng/mL in the 100-mg QD group. Exposure at steady state varied widely among patients (coefficient of variation for *C*_max_ and AUC was 11.3%-95.8% and 11.9%-133.1%, respectively, across the dose groups). Dose linearity was not observed within the dose range of 2-120 mg. Although signs of dose linearity were observed in the range of 2-80 mg (Supplementary Fig. S4), further analysis using the power model (*Y* = *α* × dose *β*) yielded indeterminate findings; therefore, dose linearity has not yet been confirmed. There was no significant accumulation (as reflected by the accumulation ratio [*R*_ac_]) of senaparib after QD administration (mean *R*_ac_ = 1.1-1.5) and only slight accumulation after BID administration (*R*_ac_ = 1.8).

### Preliminary Efficacy

#### ITT Population

**Figure 2.**
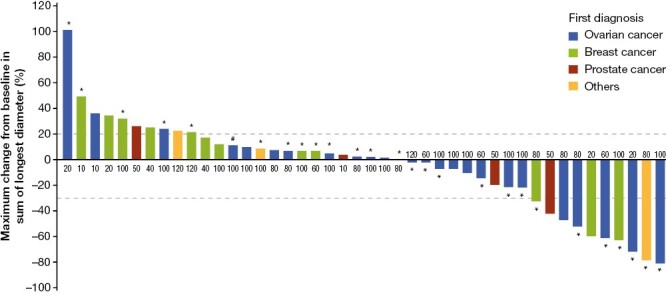
Waterfall plot of best change in target lesions in the evaluable ITT population (dose escalation and dose expansion) showing tumor type and BRCA^mut+^ status. The numbers at the x axis denote the dose cohort of the patient represented by the associated bar (10-120 mg QD or 50 mg BID). *Patients harboring BRCA^mut+^ tumors. ^†^Patient with ovarian cancer and double primary breast cancer. BID, twice daily; BRCA^mut+^, patients harboring mutations in *BRCA1* or *BRCA2*; ITT, intent to treat; QD, once daily.

Forty-four (77.2%) patients in the ITT population with target lesions at baseline and at least one imaging examination after treatment were evaluable for efficacy (Table 3). Ten patients experienced PR (there were no CRs), for an ORR of 22.7%. Eighteen (40.9%) patients overall achieved stable disease, providing a DCR of 63.6%. The ORRs for patients with ovarian, breast, or prostate cancer were comparable (5/25 [20.0%], 3/12 [25.0%], and 1/4 [25.0%], respectively), while a greater proportion of patients with ovarian cancer experienced a decrease from baseline in tumor size compared with non-ovarian cancers (13/24 [54.2%] vs. 6/20 [30.0%]). In addition, the percentage of patients with a reduction from baseline in tumor size was higher among BRCA^mut+^ patients than for their BRCA wild-type counterparts (13/26 [50.0%] vs. 6/18 [33.3%]) (Fig. 2). Median DOR was 169 days (95% CI, 1-not reached [NR]). Median PFS for the ITT population was 167 days (95% CI, 56-255 days).

**Table 3.** Tumor response to senaparib and evaluation of clinical benefit (dose-escalation and dose-expansion periods, ITT populations; *n* = 44 of 57 evaluable for response)

Efficacy indicator (ITT)	Dose groups										
	2 mg QD *n* = 1	5 mg QD *n* = 3	10 mg QD *n* = 3	20 mg QD *n* = 5	40 mg QD *n* = 3	60 mg QD *n* = 5	80 mg QD *n* = 9	100 mg QD *n* = 20	120 mg QD *n* = 4	50 mg BID *n* = 4	Total *N* = 57
Evaluable, *n*	0	0	3	4	2	4	8	17	3	3	44
ORR,^a^***n* (%)**	0	0	0 (0)	2 (50.0)	0 (0)	1 (25.0)	4 (50.0)	2 (11.8)	0 (0)	1 (33.3)	10 (22.7)
(95% CI)											
** Complete response**	–	—	0	0	0	0	0	0	0	0	0
** Partial response**	—	—	0	2 (50.0)	0	1 (25.0)	4 (50.0)	2 (11.8)	0	1 (33.3)	10 (22.7)
** Stable disease** ^b^	—	—	1 (33.3)	0	0	3 (75.0)	2 (25.0)	10 (58.8)	1 (33.3)	1 (33.3)	18 (40.9)
DCR,^c^***n* (%)**	—	—	1 (33.3)	2 (50.0)	0 (0)	4 (100)	6 (75.0)	12 (70.6)	1 (33.3)	2 (66.7)	28 (63.6)
(95% CI)	—	—	(0.8-90.6)	(6.8-93.2)	—	(39.8-100)	(34.9-96.8)	(44.0-89.7)	(0.8-90.6)	(9.4-99.2)	(47.8-77.6)
DOR**, days**											
Evaluable, *n*	0	0	0	2	0	1	4	2	0	1	44
** Events, *n* (%)**	0	0	0	1 (50.0)	0	0	4 (100)	1 (50.0)	0	0	6 (60.0)
** Median (95% CI)** ^d^	—	—	—	— (1-NR)	—	—	144.5 (39-211)	— (169-NR)	—	—	169 (1-NR)
PFS**, days**											
Evaluable, *n*	1	3	3	5	3	5	9	20	4	4	57
** Events, *n* (%)**	0 (0)	2 (66.7)	3 (100)	3 (60.0)	2 (66.7)	2 (40.0)	8 (88.9)	8 (40.0)	3 (75.0)	1 (25.0)	32 (56.1)
** Median (95% CI)** ^e^	—	464 (45-464)	49 (47-88)	78 (39-NR)	49 (48-NR)	255 (127-NR)	167 (40-216)	289 (48-NR)	153 (44-258)	— (46-NR)	167 (56-255)

^a^ORR, complete response +partial response.

^b^A best overall response of stable disease was confirmed only if the tumor imaging date of the visit at which stable disease was recorded was ≥42 days after the first dose of senaparib (cycle 1 day 1).

^c^DCR, complete response+partial response+stable disease.

^d^95% CI calculated using the accurate probability method.

^e^95% CI calculated using the Kaplan-Meier method.

Abbreviations: BID, twice daily; C1, confidence interval; DCR, disease control rate; DOR, duration of response; ITT, intent to treat; NR, not reached; ORR, objective response rate; PFS, progression-free survival; QD, once daily.

#### BRCA^mut+^ Population

In the subgroup of evaluable BRCA^mut+^ patients (*n* = 32), 26 were evaluable for tumor response. Seven patients achieved PR, for an ORR of 26.9%, varying across the QD dose groups from 0% to 50.0% (Supplementary Table S8). There were no evaluable BRCA^mut+^ patients in the 50-mg BID dose group. Disease control was achieved by 19 patients (including 12 with stable disease), for a DCR of 73.1%. As in the ITT population, a greater proportion of patients with ovarian cancer in the BRCA^mut+^ subgroup experienced a reduced tumor size compared with their non-ovarian counterparts (10/16 [62.5%] vs. 3/10 [30.0%]) (Supplementary Fig. S5). Median DOR in BRCA^mut+^ patients was 169 days (95% CI, 39-NR). Median PFS was 215 days (95% CI, 79-464).

## Discussion

Senaparib was well tolerated, had good PK properties, and showed encouraging signs of antitumor activity in Chinese patients with previously treated, advanced solid tumors, as well as in a subpopulation of patients harboring BRCA^mut+^. Exposure to senaparib increased with increasing doses in the range of 2-80 mg, with a trend toward absorption saturation at higher doses, and with insignificant levels of accumulation. Based on the safety, PK, and efficacy data, the RP2D for the Chinese phase II study was determined to be 100 mg QD; however, the observation of antitumor activity and the lack of dose-limiting toxicities in the dose range of 20-100 mg QD indicate a potentially wide therapeutic window.

The safety profile of senaparib in the Chinese patients in this study was generally consistent with those of currently approved PARP inhibitors,^19,20^ with no unexpected safety signals.^21^ Rates of grade ≥3 nonhematologic toxicity were low. The majority of TEAEs were manageable and had returned to normal, baseline, or stable status by the end of the study. The most frequent TRAEs (summarized by preferred term) were hematologic, and most were mild in severity (grade ≤3 in >90% of patients). Hematologic toxicities are a known class effect of PARP inhibitors that may be attributable to PARP-trapping-induced bone marrow suppression,^19,20,22^ and are not thought to impact the efficacy of PARP inhibitors when administered as single agents.^22^ A small but increased risk of secondary acute myeloid leukemia or myelodysplastic syndrome (AML/MDS) has been reported with PARP inhibitors; the United States Food and Drug Administration labels for olaparib, rucaparib, niraparib, and talazoparib carry a warning regarding that risk.^23-26^ There were no cases of secondary hematologic tumors in the present study population. However, since the onset of AML/MDS associated with PARP inhibition was found to be almost 18 months since the first treatment,^27^ further studies with larger patient numbers and longer follow-up durations are needed to clarify the risk of hematologic malignancy with senaparib. Additional adverse events of concern that have been reported with exposure to olaparib are pneumonitis and (for patients with metastatic castration-resistant prostate cancer) venous thromboembolic events,^23^ neither of which were observed in the present study, although there was one case of dyspnea.

PARP inhibitors have demonstrated efficacy in a variety of solid tumors, with particular benefits in tumors harboring alterations in DNA-damage repair genes, such as BRCA^mut+^.^1,13,28,29^ The preliminary efficacy data are consistent with these findings, although data should be interpreted with caution because of the limited patient numbers. Compared with the ITT population, patients harboring BRCA^mut+^ tumors had a greater reduction in tumor burden, as reflected by a higher ORR and DCR, and had a longer median PFS. The inclusion of multiple types of solid tumor in the present phase I study allowed preliminary identification of those with particular sensitivity to senaparib. Compared with other solid tumor types, a numerically greater reduction in tumor burden was observed among patients with ovarian cancer in the ITT population, and in the BRCA^mut+^ subgroup.

PARP-inhibitor-based combination therapies, including chemotherapy, other targeted therapies, and radiotherapy, are currently under investigation for solid tumors, toward achieving an additive or synergistic outcome.^30-32^ For example, evidence suggests that PARP inhibitors act synergistically with immune checkpoint blockade to inhibit tumor growth by enhancing tumor mutational burden and programmed death ligand 1 expression.^30,33^ The preliminary signs of antitumor efficacy observed herein support testing of senaparib as part of a combination therapy.

The single-arm, phase I, dose-escalation/expansion design of this study represents a limitation of this analysis. The small number of patients was not evenly distributed across the dose groups, there was no comparator arm, and much of the data was obtained at doses other than the RP2D. In addition, most of the patients had advanced tumors after failure of multiple lines of treatment and were not screened for tumor markers.

## Conclusions

Senaparib was well tolerated and showed antitumor activity, with particular benefit being observed in patients with BRCA^mut+^ disease. The findings suggest that further study is warranted in patients with advanced solid tumors, including those harboring BRCA^mut+^. Review of the safety, efficacy, and PK findings indicate that the RP2D for clinical study in China should be 100 mg QD.

## Supplementary Material

oyad163_suppl_Supplementary_Figures

oyad163_suppl_Supplementary_Tables

## Data Availability

The data underlying this article will be shared upon reasonable request to the corresponding author.
