# Supplementary material for: Safety, Tolerability, and Pharmacokinetics of Senaparib, a Novel PARP1/2 Inhibitor, in Chinese Patients With Advanced Solid Tumors: A Phase I Trial
Source: Oncologist. 2023 Jun 20;28(12):e1259–67. doi: 10.1093/oncolo/oyad163 (PMC10712727; doi:10.1093/oncolo/oyad163)
Supplement: oyad163_suppl_Supplementary_Figures [file oyad163_suppl_supplementary_figures.docx]

Safety, Tolerability, and Pharmacokinetics of Senaparib, a Novel PARP1/2 Inhibitor, in Chinese Patients with Advanced Solid Tumors: A Phase I Trial

Junning Cao, Hongqian Guo, Dongmei Ji, Weina Shen, Shun Zhang, Chih-Yi Hsieh, Sui Xiong Cai, Ye Edward Tian, Jun Bao, Ning Ma, Chen Wang, Ming Zhang, Baoyue Li, Mingchuan Guo, Ruiyu Zhou, Xiaozhu Wang, Cong Xu, Binghe Xu

Supplementary Material

## **Figure S1.** Study design. Dose-escalation (according to a modification of the Fibonacci method^1^) and dose-expansion periods.


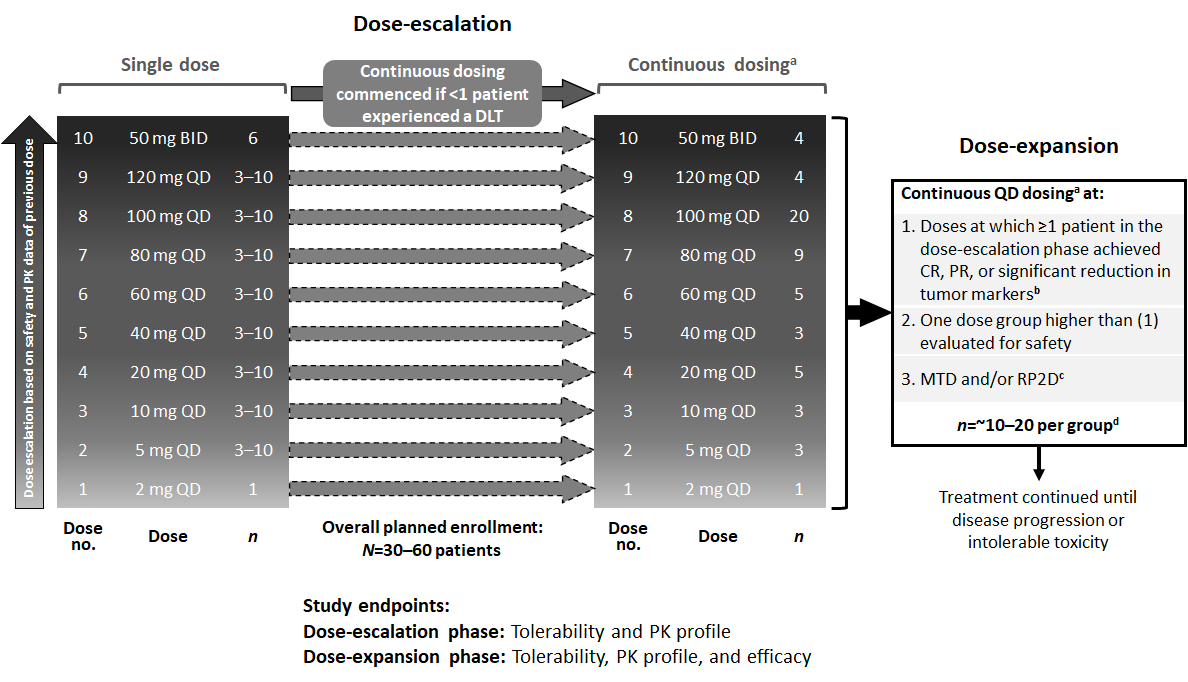


**^a^**Senaparib was administered on days 1-21 of each 3-week cycle.
**^b^**CR or PR were assessed using RECIST v1.1; tumor markers were also analyzed to determine best response in patients with prostate cancer (PSA levels) and ovarian cancer (CA-125 levels).
**^c^**Including patients from the dose-escalation phase who received the MTD/RP2D. The MTD was defined as the maximum dose at which ≤1/6 patients experienced a DLT during the first treatment cycle. The RP2D was determined based on efficacy, safety, and PK data.
**^d^**Including patients from the respective dose-escalation group plus 10 additional patients with BRCA^mut+^ solid tumors (excluding ovarian cancer, fallopian tube cancer, and primary peritoneal cancer).

BID, twice daily; BRCA^mut+^, harboring mutations in *BRCA1* and/or *BRCA2*; CA-125, carcinoembryonic antigen-125; CR, complete response; DLT, dose-limiting toxicity; MTD, maximum tolerated dose; PK, pharmacokinetic; PR, partial response; PSA, prostate-specific antigen; QD, once daily; RECIST v.1., Response Evaluation Criteria in Solid Tumors version 1.1; RP2D, recommended phase II dose.

## **Figure S2.** Mean plasma concentration-time curve for the dose-escalation, single-dose administration period. (A) Linear. (B) Semilog. PK analysis set.

**(A)**

**
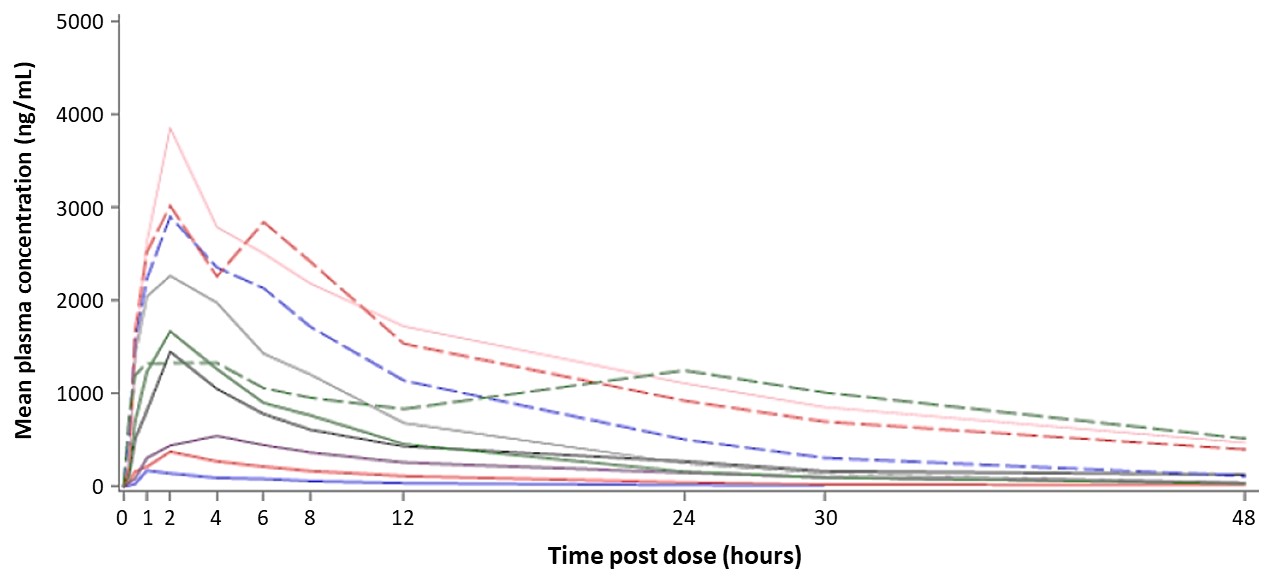
**

**(B)**

**
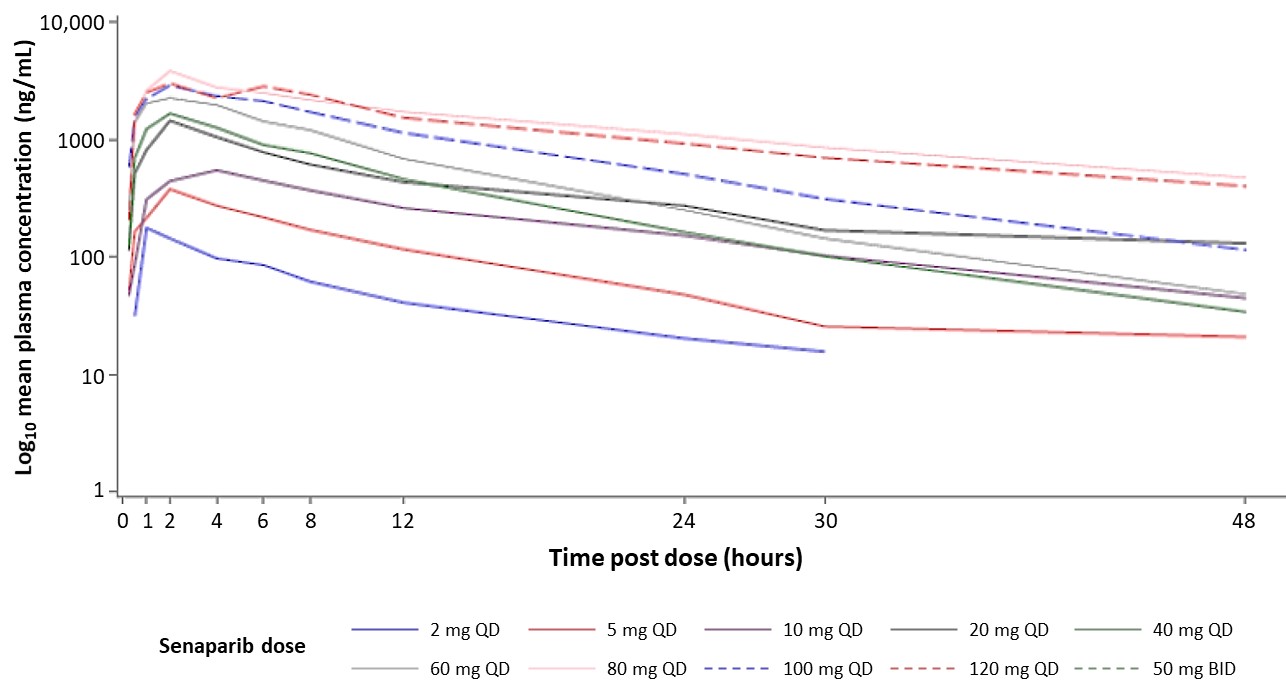
**

BID, twice daily; PK, pharmacokinetic; QD, once daily.

## **Figure S3.** Mean plasma concentration-time curve for the dose-escalation and dose-expansion continuous-dosing period (D1 and D15). (A) Linear D1 and D15. (B) Semilog C1D1. (C) Semilog C1D15. PK analysis set.

**(A)**

**
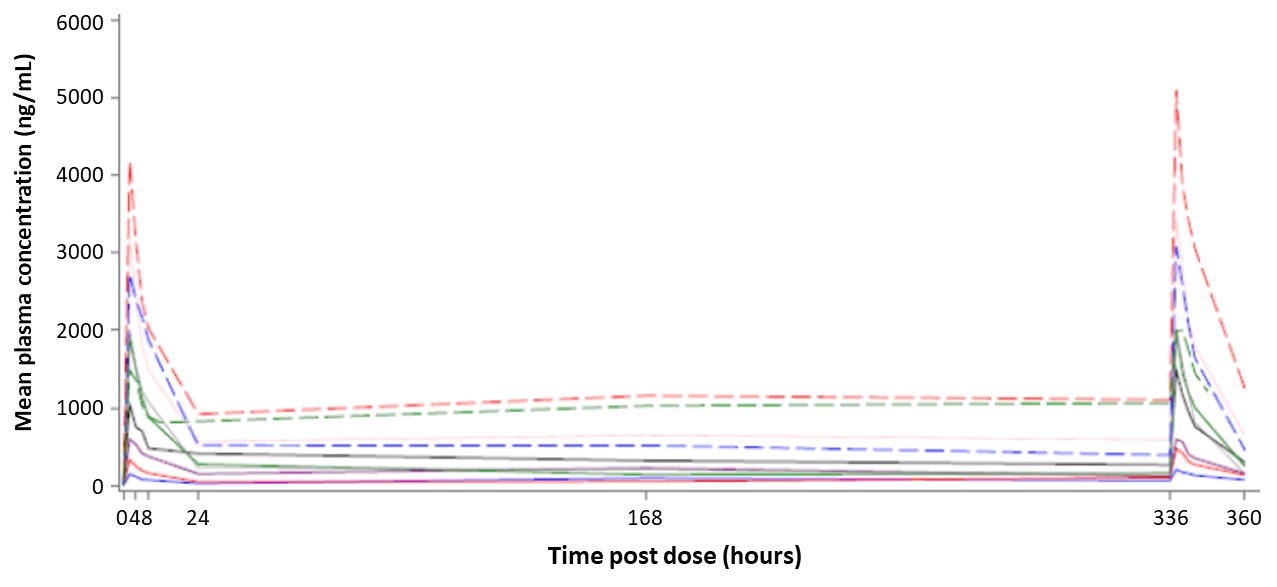
**

**(B)**

**
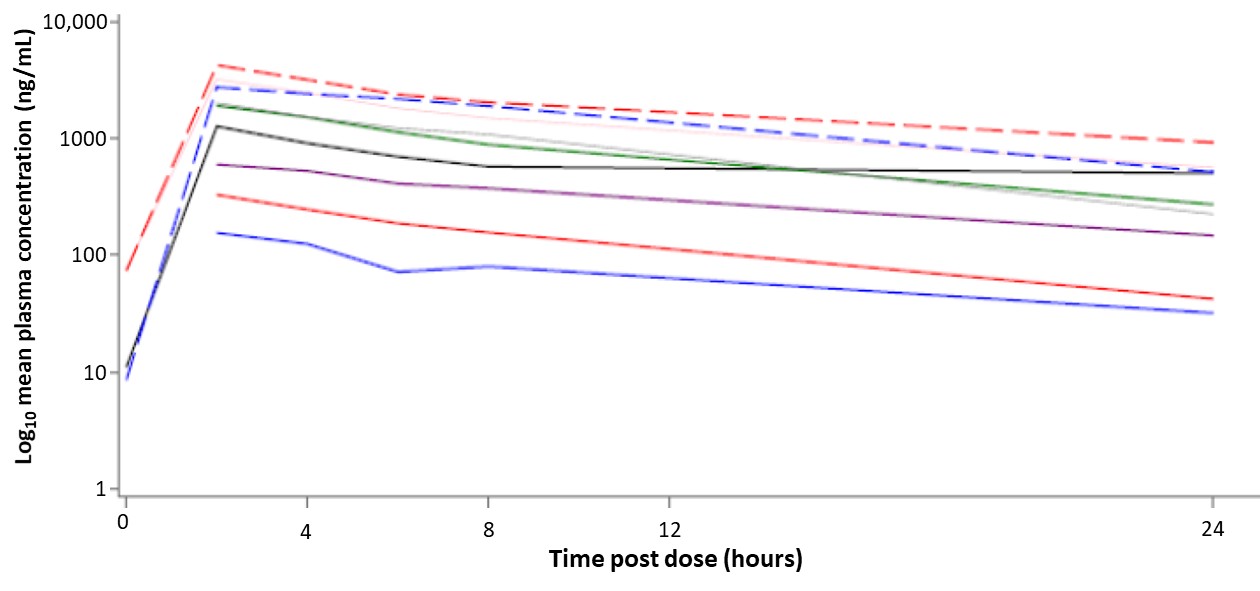
**

**(C)**


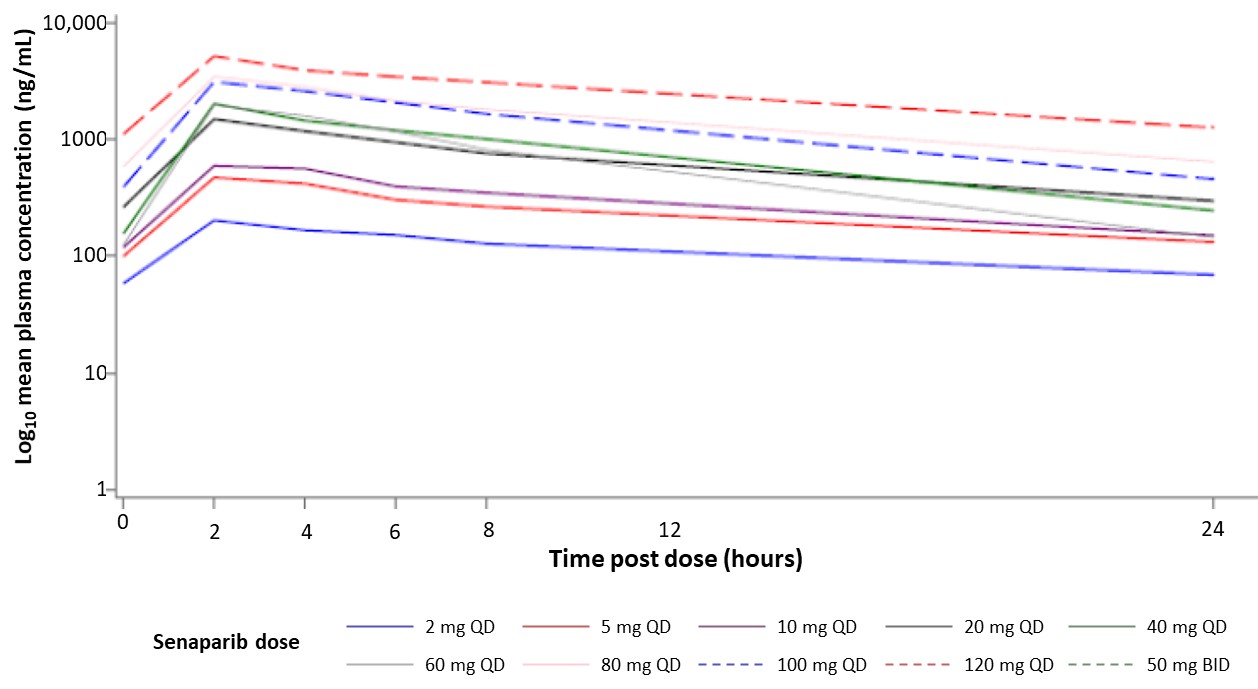


BID, twice daily; C1, cycle 1; D1, day 1; D15, day 15; PK, pharmacokinetic; QD, once daily.

## **Figure S4.** Scatter plot of dose versus AUC_0-t_ of senaparib at C1D15. PK analysis set.


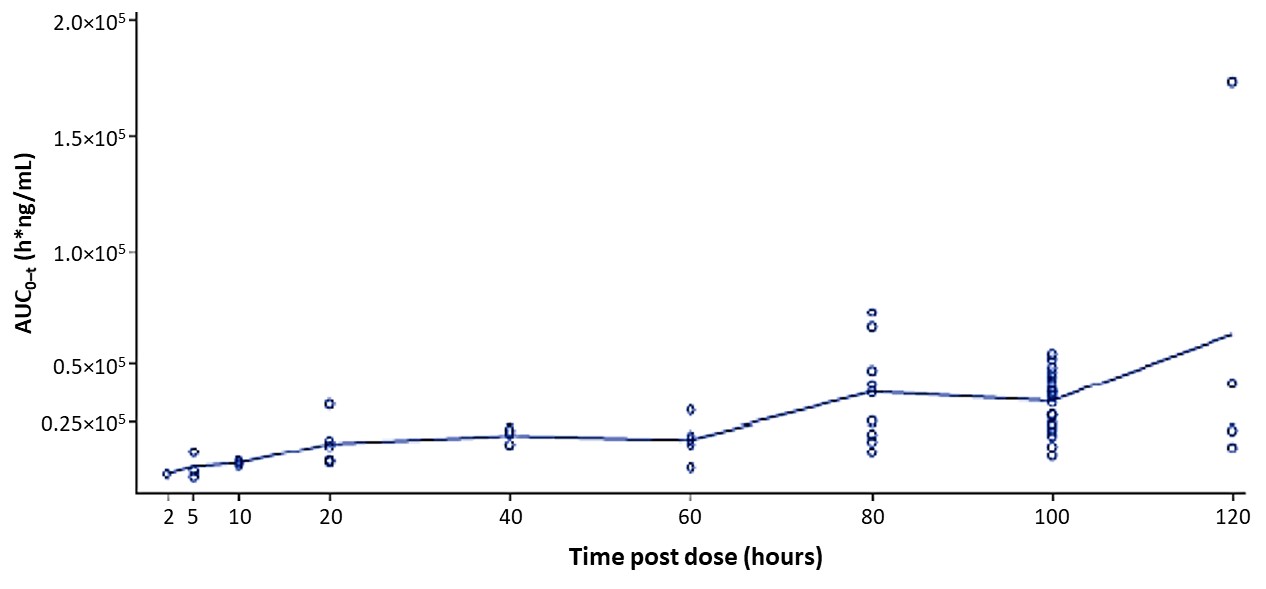


AUC_0-t_, area under the time–concentration curve from time 0 to the last measurable plasma concentration; C1D15, day 15 of cycle 1; PK, pharmacokinetic.

## **Figure S5.** Waterfall plot of best change in target lesions in the evaluable population of patients with BRCA^mut+^ tumors (dose-escalation and dose-expansion) showing tumor type and dose group.

**
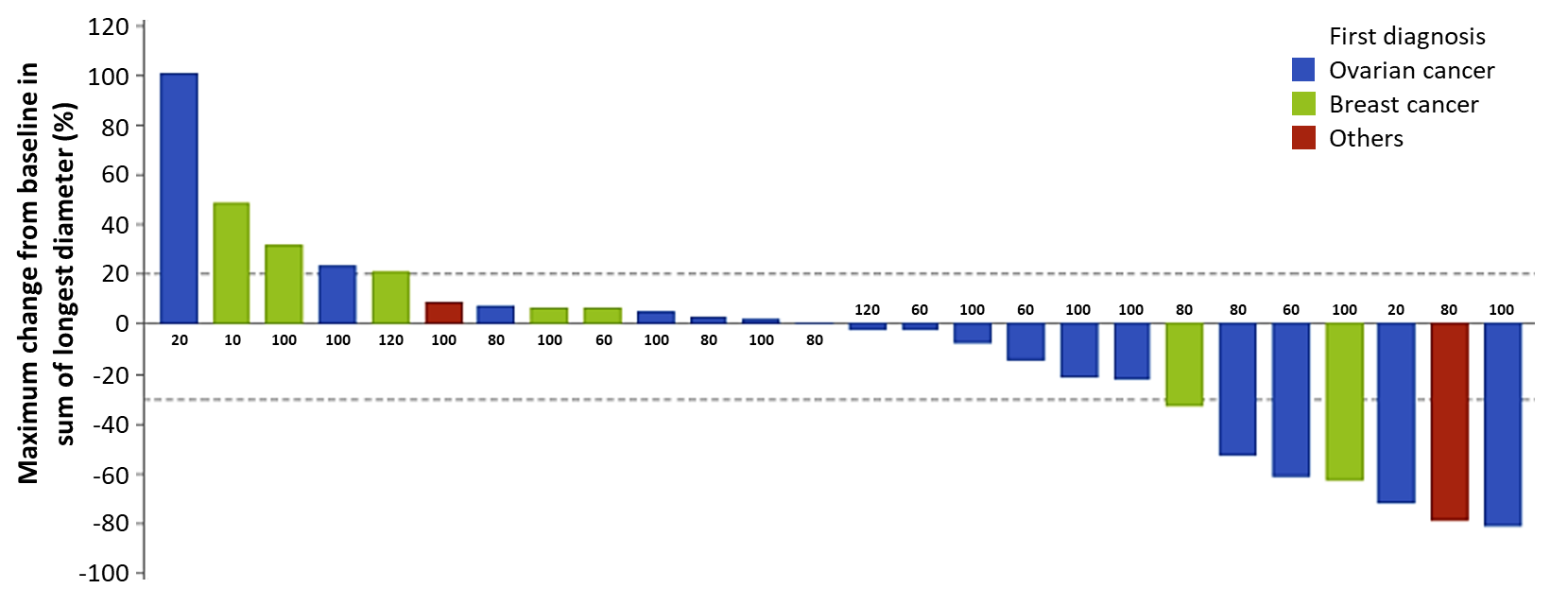
**

The numbers at the x axis denote the dose cohort of the patient represented by the associated bar (10 mg-120 mg QD).

BRCA^mut+^, patients harboring mutations in *BRCA1* or *BRCA2*; QD, once daily.

# Supplementary References

1. Le Tourneau C, Lee JJ, Siu LL. Dose escalation methods in phase I cancer clinical trials. *J Natl Cancer Inst.* 2009;101(10):708-720. <https://doi.org/10.1093/jnci/djp079>.
