# Supplementary material for: Safety, Tolerability, and Pharmacokinetics of Senaparib, a Novel PARP1/2 Inhibitor, in Chinese Patients With Advanced Solid Tumors: A Phase I Trial
Source: Oncologist. 2023 Jun 20;28(12):e1259–67. doi: 10.1093/oncolo/oyad163 (PMC10712727; doi:10.1093/oncolo/oyad163)
Supplement: oyad163_suppl_Supplementary_Tables [file oyad163_suppl_supplementary_tables.docx]

Safety, Tolerability, and Pharmacokinetics of Senaparib, a Novel PARP1/2 Inhibitor, in Chinese Patients with Advanced Solid Tumors: A Phase I Trial

Junning Cao, Hongqian Guo, Dongmei Ji, Weina Shen, Shun Zhang, Chih-Yi Hsieh, Sui Xiong Cai, Ye Edward Tian, Jun Bao, Ning Ma, Chen Wang, Ming Zhang, Baoyue Li, Mingchuan Guo, Ruiyu Zhou, Xiaozhu Wang, Cong Xu, Binghe Xu

Supplementary Material

# Supplementary Methods

## Dose Escalation

After the initial single administration of senaparib, the dose was sequentially escalated according to the modified Fibonacci method (**Figure S1;** **Table S1**). The initial dose was 2 mg orally once daily (QD), which was administered to one patient to observe safety. The proposed dose escalation included the following: 5 mg, 10 mg, 20 mg, 40 mg, 60 mg, 80 mg, 100 mg, and 120 mg. The next dose was determined by the investigator/sponsor based on the safety data and pharmacokinetic (PK) profile of the previous dose group. If there was no dose limiting toxicity (DLT) in a dose group, the patient proceeded to the next dose group until a DLT occurred. If one third of patients in a particular dose group experienced a DLT, three additional patients were enrolled to the same group. If no DLT occurred in these three additional patients, they entered the next dose group. Dose escalation was stopped if ≥1 of the additional three patients, or one third of all patients, experienced a DLT. Simultaneously, three additional patients were enrolled to the previous dose group, until the maximum tolerated dose (MTD) and/or recommended phase II dose (RP2D) were determined. If all patients in a dose group experienced grade 2 nonhematologic toxicity or grade 3 hematologic toxicity, the investigator and the sponsor determined the next dose after reviewing the clinical safety data and PK data. The MTD dose group required at least six subjects for confirmation. In the absence of an MTD, the manner of dose escalation and whether dose escalation was to be terminated was decided by the sponsor together with the investigator, based on the efficacy data (eg, objective response rate [ORR], duration of response [DOR], disease control rate [DCR]), safety data (eg, grade, frequency, duration of senaparib-related and/or senaparib-unrelated adverse events), and PK data (eg, saturation of maximum plasma concentration [C_max_], area under the time-concentration curve [AUC], minimum plasma concentration [C_min_]).

A twice-daily (BID) 50-mg dose group was added based on the tolerability, efficacy, and PK profile findings for the single-dose administrations.

## **Table S1.** Study eligibility criteria

| **Inclusion criteria** |
| --- |
| - Signed Informed Consent Form |
| - Age ≥18 years |
| - Histologically or cytologically documented disease: incurable advanced solid malignancy that had progressed on, or failed to respond to, at least one prior system therapy |
| - ECOG PS of 0 or 1 |
| - In the dose-expansion stage, patients with a BRCA mutation were enrolled; patients with breast cancer, ovarian cancer, and prostate cancer were preferred - In the dose-escalation period, there was at least one evaluable lesion; in the dose-expansion period, there was at least one measurable lesion according to RECIST v1.1 criteria (at least one evaluable lesion was acceptable for prostate cancer) |
| **Exclusion criteria** |
| - Hematologic or organ dysfunction, defined by the following (hematologic parameters were assessed ≥14 days after a prior treatment, if any): - ANC <1.5×10^9^/L - Hemoglobin <9 g/dL - Platelet count <100×10^9^/L - Total bilirubin >1.5×ULN, or >2.5×ULN in patients with documented liver metastases - AST and/or ALT >2.5×ULN, or >3×ULN in patients with documented liver metastases - Serum creatinine >1.5×ULN - INR >1.5×ULN or aPTT >1.5×ULN (INR only for patients who did not receive therapeutic anticoagulation) |
| - Received chemotherapy, biologic therapy, radiotherapy, and other antitumor therapy ≤4 weeks before treatment, or endocrine therapy (except necessary medical castration therapy for patients with mCRPC) or small-molecule targeted therapy ≤2 weeks before treatment |
| - AEs from prior anticancer therapy that had not resolved to NCI CTCAE grade ≤1, except for alopecia |
| - Previous treatment with drugs targeting PARP |
| - Clinically significant active infection |
| - Known clinically significant history of liver disease, including viral or other hepatitis, current alcohol abuse, or cirrhosis (except for patients with previous viral hepatitis confirmed by PCR testing who are not in the active stage) |
| - Known human immunodeficiency virus infection |
| - New York Heart Association Class II or greater congestive heart failure; history of myocardial infarction or unstable angina within 6 months prior to day 1; history of stroke or transient ischemic attack within 6 months prior to day 1 |
| - Active or untreated brain metastasis |
| - Pregnancy (positive pregnancy test) or lactating women |
| - Patients with fertility and their spouses are unwilling to accept effective contraceptive measures during treatment and ≤90 days after the last dose |
| - Patients with dysphagia or oral malabsorption |
| - Patients who, in the investigator’s opinion, have poor adherence, or any factors that prevent them from participating in the trial |
| - The investigator believes that the patient has any clinical or laboratory abnormalities and is not suitable for this clinical study |
| AE, adverse event; ALT, alanine aminotransferase; ANC, absolute neutrophil count; aPTT, activated partial thromboplastin time; AST, aspartate aminotransferase; ECOG PS, Eastern Cooperative Oncology Group performance status; INR, international normalized ratio; mCRPC, metastatic castration-resistant prostate cancer; NCI CTCAE, National Cancer Institute Common Terminology Criteria for Adverse Events; PARP, poly(ADP-ribose) polymerase; PCR, polymerase chain reaction; RECIST v1.1, Response Evaluation Criteria in Solid Tumors version 1.1; ULN, upper limit of normal. |

## **Table S2.** Study analysis populations

| **Analysis population** | **Definition** | **Data analyzed** |
| --- | --- | --- |
| Safety analysis set | All enrolled patients who received ≥1 dose of senaparib and underwent at least one safety evaluation | All safety data (eg, TEAEs, TRAEs, SAEs, laboratory tests, vital signs, physical examinations, 12-lead ECGs) except DLTs, and baseline and demographic data |
| DLT analysis set | All enrolled patients who experienced a DLT during the first cycle in the dose-escalation period and those who did not experience a DLT but completed the first cycle of treatment in the dose-escalation period  DLTs were defined as any of the following toxicities (graded according to NCI CTCAE v4.03^1^) occurring during the period C1D1–C1D21:   - Any grade ≥3 nonhematologic toxicity (nausea, vomiting, diarrhea, constipation, and electrolyte imbalance grade ≥3 after symptomatic and supportive treatment) - Grade 4 anemia - Grade 4 neutropenia - Grade 3 granulocytopenia with fever - Grade 4 thrombocytopenia or grade 3 thrombocytopenia accompanied by hemorrhage - Dose interruption for >14 days due to toxicity | DLTs during the dose-escalation period |
| PK analysis set | All enrolled patients who received ≥1 dose of senaparib and had ≥1 post-dose PK evaluation | All PK parameters, according to frequency of administration (ie, single vs continuous) and dose |
| Efficacy analysis set (ITT) | All patients who received ≥1 dose senaparib and who had target lesions at baseline and at least one imaging examination after treatment; subgroup analyses were conducted for patients with BRCA^mut+^ tumors and according to CA-125 status (ovarian cancer) and PSA response (ie, PSA decrease of ≥50%) | CR, PR, ORR, DCR, DOR (in patients with a response), and PFS |
| BRCA^mut+^, tumor harboring *BRCA1* and/or *BRCA2* mutations; C1D1, cycle 1, day 1; C1D21, cycle 1, day 21; CA-125, carcinoembryonic antigen; CR, complete response; DCR, disease control rate; DLT, dose-limiting toxicity; DOR, duration of response; ECG, electrocardiogram; ITT, intent to treat; NCI CTCAE v4.03, National Cancer Institute Common Terminology Criteria for Adverse Events version 4.03; ORR, objective response rate; PFS, progression-free survival; PK, pharmacokinetic; PR, partial response; PSA, prostate-specific antigen; SAEs, serious adverse events; TEAEs, treatment-emergent adverse events; TRAEs, treatment (senaparib)-related adverse events. | | |

# Supplementary Results

## **Table S3.** Study drug administration (dose-escalation and dose-expansion periods)

| Characteristic | Dose group | | | | | | | | | | Total  *N =*57 |
| --- | --- | --- | --- | --- | --- | --- | --- | --- | --- | --- | --- |
|  | **2 mg QD**  ***n* = 1** | **5 mg QD**  ***n* = 3** | **10 mg QD**  ***n* = 3** | **20 mg QD**  ***n* = 5** | **40 mg QD**  ***n* = 3** | **60 mg QD**  ***n* = 5** | **80 mg QD**  ***n* = 9** | **100 mg QD**  ***n* = 20** | **120 mg QD**  ***n* = 4** | **50 mg BID**  ***n* = 4** |  |
| Exposure (mg)^a^ | | | | | | | | | | | |
| *n* (*n*miss) | 1 (0) | 3 (0) | 3 (0) | 5 (0) | 3 (0) | 5 (0) | 9 (0) | 20 (0) | 4 (0) | 4 (0) | 57 (0) |
| Mean ±SD | 44.0 ± – | 2243.3 ±3337.2 | 570.0 ±242.5 | 2232.4 ±2155.1 | 2346.7 ±1051.0 | 10 704.0 ±8234.2 | 9028.9 ±6496.1 | 14 658.0 ±14 730.4 | 11 280.0 ±12 848.4 | 6637.5 3305.4 | 9233.3 ±11 011.1 |
| Median | 44 | 420 | 430 | 1040 | 1760 | 7740 | 9260 | 7040 | 5640 | 5375 | 4400 |
| (Q1, Q3) | (44.0, 44.0) | (215.0, 6095.0) | (430.0, 850.0) | (880.0, 2522.0) | (1720.0, 3560.0) | (5220.0, 15 180.0) | (3520.0, 13 520.0) | (4200.0, 24 590.0) | (4320.0, 18 240.0) | (4400.0, 8875.0) | (3200.0, 11 400.0) |
| Min-max | 44-44 | 2-156 095 | 430-850 | 8-405 880 | 17-203 560 | 258-22 800 | 176-20 160 | 320-57 600 | 336-30 480 | 440-11 400 | 4-457 600 |
| Relative dose intensity,^a,b^ *n* (%) | | | | | | | | | | | |
| <80% | 0 | 2 (66.7) | 2 (66.7) | 2 (40.0) | 2 (66.7) | 1 (20.0) | 5 (55.6) | 12 (60.0) | 3 (75.0) | 3 (75.0) | 32 (56.1) |
| 80%-120% | 1 (100) | 0 | 1 (33.3) | 3 (60.0) | 1 (33.3) | 4 (80.0) | 4 (44.4) | 8 (40.0) | 1 (25.0) | 1 (25.0) | 24 (42.1) |
| >120% | 0 | 1 (33.3) | 0 | 0 | 0 | 0 | 0 | 0 | 0 | 0 | 1 (1.8) |
| Duration of senaparib administration (days)^a,c^ | | | | | | | | | | | |
| *n* (*n*miss) | 1 (0) | 3 (0) | 3 (0) | 5 (0) | 3 (0) | 5 (0) | 9 (0) | 20 (0) | 4 (0) | 4 (0) | 57 (0) |
| Mean ±SD | 22.0 ±– | 196.7 ±231.6 | 57.0 ±24.3 | 111.8 ±107.8 | 58.7 ±26.3 | 178.4 ±137.2 | 119.4 ±80.7 | 149.7 ±149.7 | 94.0 ±107.1 | 88.5 33.0 | 126.5 ±122.2 |
| Median | 22 | 84 | 43 | 52 | 44 | 129 | 125 | 74 | 47 | 76 | 66 |
| (Q1, Q3) | (22.0, 22.0) | (43.0, 463.0) | (43.0, 85.0) | (44.0, 127.0) | (43.0, 89.0) | (87.0, 253.0) | (44.0, 169.0) | (42.0, 251.5) | (36.0, 152.0) | (66.0, 111.0) | (43.0, 169.0) |
| Min-max | 22-22 | 43-463 | 43-85 | 42-294 | 46-89 | 43-380 | 22-252 | 32-576 | 28-254 | 66-136 | 22-576 |
| ^a^For calculating exposure, relative dose intensity, and duration of study drug administration, the end times of scheduled and actual medication were involved and both were cut off on June 9, 2020. ^b^Relative dose intensity = (actual dose administered by subject/dose specified by subject protocol) × 100%. ^c^Duration of study drug administration (days) = last time of study drug administration - first time of study drug administration + 1 (excluding the time of drug interruption during the trial). For one patient, the relative dose intensity was >120%, and their senaparib dose was increased from 5 mg to 20 mg on July 26, 2018 with Ethics Committee approval. BID, twice daily; Max, maximum; Min, minimum; *n* (*n*miss), number of patients with data (number of patients with missing data); Q1, Q3, interquartile range; QD, once daily. | | | | | | | | | | | |

## **Table S4.** Summary of treatment-emergent adverse events

|  | Dose group | | | | | | | | | | | | | | | | | | | | Total  *N* = 57 | |
| --- | --- | --- | --- | --- | --- | --- | --- | --- | --- | --- | --- | --- | --- | --- | --- | --- | --- | --- | --- | --- | --- | --- |
|  | **2 mg QD**  ***n* = 1** | | **5 mg QD**  ***n* = 3** | | **10 mg QD**  ***n* = 3** | | **20 mg QD**  ***n* = 5** | | **40 mg QD**  ***n* = 3** | | **60 mg QD**  ***n* = 5** | | **80 mg QD**  ***n* = 9** | | **100 mg QD**  ***n* = 20** | | **120 mg QD**  ***n* = 4** | | **50 mg BID^a^**  ***n* = 4** | |  |  |
|  | ***n* (%)** | ***n* ev** | ***n* (%)** | ***n* ev** | ***n* (%)** | ***n* ev** | ***n* (%)** | ***n* ev** | ***n* (%)** | ***n* ev** | ***n* (%)** | ***n* ev** | ***n* (%)** | ***n* ev** | ***n* (%)** | ***n* ev** | ***n* (%)** | ***n* ev** | ***n* (%)** | ***n* ev** | ***n* (%)** | ***n* ev** |
| Any TEAE | 1 (100) | 9 | 3 (100) | 28 | 3 (100) | 10 | 5 (100) | 30 | 3 (100) | 7 | 5 (100) | 33 | 9 (100) | 132 | 20 (100) | 158 | 4 (100) | 52 | 4 (100) | 32 | 57 (100) | 491 |
| Grade ≥3 | 1 (100) | 1 | 3 (100) | 5 | 0 | 0 | 2 (40.0) | 3 | 0 | 0 | 2 (40.0) | 2 | 6 (66.7) | 12 | 7 (35.0) | 9 | 3 (75.0) | 6 | 3 (75.0) | 3 | 27 (47.4) | 41 |
| TRAEs | 1 (100) | 6 | 3 (100) | 13 | 3 (100) | 1 | 3 (60.0) | 15 | 3 (100) | 6 | 5 (100) | 27 | 9 (100) | 109 | 20 (100) | 144 | 4 (100) | 47 | 4 (100) | 26 | 55 (96.5) | 403 |
| Grade ≥3 | 0 | 0 | 1 (33.3) | 1 | 0 | 0 | 1 (20.0) | 1 | 0 | 0 | 0 | 0 | 5 (55.6) | 10 | 7 (35.0) | 9 | 3 (75.0) | 6 | 2 (50.0) | 2 | 19 (33.3) | 29 |
| SAEs | 1 (100) | 1 | 0 | 0 | 0 | 0 | 1 (20.0) | 1 | 0 | 0 | 2 (40.0) | 2 | 5 (55.6) | 5 | 1 (5.0) | 1 | 1 (25.0) | 1 | 1 (25.0) | 1 | 12 (21.1) | 12 |
| TEAEs leading to: | |  |  |  |  |  |  |  |  |  |  |  |  |  |  |  |  |  |  |  |  |  |
| Dose discontinuation | 0 | 0 | 0 | 0 | 0 | 0 | 2 (40.0) | 2 | 0 | 0 | 1 (20.0) | 1 | 1 (11.1) | 4 | 1 (5.0) | 1 | 1 (25.0) | 2 | 0 | 0 | 6 (10.5) | 10 |
| Dose interruption | 0 | 0 | 0 | 0 | 0 | 0 | 1 (20.0) | 1 | 0 | 0 | 1 (20.0) | 1 | 4 (44.4) | 9 | 6 (30.0) | 8 | 2 (50.0) | 3 | 1 (25.0) | 1 | 15 (26.3) | 23 |
| Dose reduction | 0 | 0 | 0 | 0 | 0 | 0 | 0 | 0 | 0 | 0 | 0 | 0 | 3 (33.3) | 3 | 4 (20.0) | 6 | 0 | 0 | 1 (25.0) | 1 | 8 (14.0) | 10 |
| Death | 0 | 0 | 0 | 0 | 0 | 0 | 1 (20.0) | 1 | 0 | 0 | 0 | 0 | 1 (11.1) | 1 | 0 | 0 | 0 | 0 | 0 | 0 | 2 (3.5) | 2 |
| ^a^Dose group added as a result of analysis of safety, PK, and efficacy of the completed QD dose groups in the dose-escalation period of the phase I Australian trial (NCT03507543).  BID, twice daily; *n* ev, number of events; PK, pharmacokinetic; QD, once daily; SAE, serious adverse event; TEAE, treatment-emergent adverse event; TRAE, treatment (senaparib)-related adverse event. | | | | | | | | | | | | | | | | | | | | | | |

Table S5. Incidence of treatment-emergent adverse events occurring in ≥10% of patients, any grade and grade ≥3, by preferred term (dose-escalation and dose-expansion phases; safety analysis set, *N* = 57)

| TRAE (by preferred term), *n* (%) | Senaparib dose group | | | | | | | | | | | | | | | | | | | | Total  *N* = 57 | |
| --- | --- | --- | --- | --- | --- | --- | --- | --- | --- | --- | --- | --- | --- | --- | --- | --- | --- | --- | --- | --- | --- | --- |
|  | **2 mg QD** *n* = 1 | | **5 mg QD**  *n* = 3 | | **10 mg QD** *n* = 3 | | **20** **mg QD**  *n* = 5 | | **40 mg QD**  *n* = 3 | | **60 mg QD**  *n* = 5 | | **80 mg** Q**D**  *n* = 9 | | **100 mg QD**  *n* = 20 | | **120 mg QD**  *n* = 4 | | **50 mg BID**  *n* = 4 | |  |  |
|  | **All** | **Gr≥3** | **All** | **Gr≥3** | **All** | **Gr≥3** | **All** | **Gr≥3** | **All** | **Gr≥3** | **All** | **Gr≥3** | **All** | **Gr≥3** | **All** | **Gr≥3** | **All** | **Gr≥3** | **All** | **Gr≥3** | **All** | **Gr≥3** |
| Any | 1 (100) | 1 (100) | 3 (100) | 3 (100) | 3 (100) | 0 | 5 (100) | 2 (40.0) | 3 (100) | 0 | 5 (100) | 2 (40.0) | 9 (100) | 6 (66.7) | 20 (100) | 7 (35.0) | 4 (100) | 3 (75.0) | 4 (100) | 3 (75.0) | 57 (100) | 27 (47.4) |
| Anemia^a^ | 0 | 0 | 2 (66.7) | 1 (33.3) | 1 (33.3) | 0 | 1 (20.0) | 1 (20.0) | 1 (33.3) | 0 | 0 | 0 | 7 (77.8) | 3 (33.3) | 11 (55.0) | 4 (20.0) | 3 (75.0) | 1 (25.0) | 3 (75.0) | 2 (50.0) | 29 (80.9) | 12 (21.1) |
| WBC decreased | 0 | 0 | 1 (33.3) | 0 | 1 (33.3) | 0 | 1 (20.0) | 0 | 0 | 0 | 2 (40.0) | 0 | 5 (55.6) | 1 (11.1) | 9 (45.0) | 1 (5.0) | 4 (100) | 0 | 2 (50.0) | 0 | 25 (43.9) | 2 (3.5) |
| Platelet count decreased | 0 | 0 | 2 (66.7) | 0 | 0 | 0 | 1 (20.0) | 0 | 0 | 0 | 2 (40.0) | 0 | 4 (44.4) | 2 (22.2) | 6 (30.0) | 1 (5.0) | 1 (25.0) | 0 | 1 (25.0) | 0 | 17 (29.8) | 3 (5.3) |
| Asthenia | 1 (100) | 0 | 2 (66.7) | 0 | 0 | 0 | 1 (20.0) | 0 | 1 (33.3) | 0 | 1 (20.0) | 0 | 2 (22.2) | 0 | 5 (25.0) | 0 | 2 (50.0) | 0 | 1 (25.0) | 0 | 16 (28.1) | 0 |
| Nausea | 0 | 0 | 1 (33.3) | 0 | 0 | 0 | 1 (20.0) | 0 | 0 | 0 | 1 (20.0) | 0 | 4 (44.4) | 0 | 4 (20.0) | 0 | 1 (25.0) | 0 | 2 (50.0) | 0 | 14 (24.6) | 0 |
| Appetite decreased | 1 (100) | 0 | 1 (33.3) | 0 | 1 (33.3) | 0 | 1 (20.0) | 0 | 0 | 0 | 1 (20.0) | 0 | 3 (33.3) | 0 | 5 (25.0) | 0 | 1 (25.0) | 0 | 0 | 0 | 14 (24.6) | 0 |
| Neutrophil count decreased | 0 | 0 | 1 (33.3) | 0 | 0 | 0 | 0 | 0 | 0 | 0 | 1 (20.0) | 0 | 4 (44.4) | 1 (11.1) | 5 (25.0) | 2 (10.0) | 1 (25.0) | 0 | 0 | 0 | 12 (21.1) | 3 (5.3) |
| Vomiting | 0 | 0 | 0 | 0 | 0 | 0 | 0 | 0 | 0 | 0 | 1 (20.0) | 1 (20.0) | 3 (33.3) | 1 (11.1) | 5 (25.0) | 0 | 1 (25.0) | 0 | 2 (50.0) | 0 | 12 (21.1) | 2 (3.5) |
| ALT increased | 0 | 0 | 0 | 0 | 1 (33.3) | 0 | 0 | 0 | 0 | 0 | 0 | 0 | 1 (11.1) | 0 | 5 (25.0) | 0 | 2 (50.0) | 1 (25.0) | 1 (25.0) | 0 | 10 (17.5) | 1 (1.8) |
| AST increased | 0 | 0 | 0 | 0 | 1 (33.3) | 0 | 0 | 0 | 0 | 0 | 0 | 0 | 2 (22.2) | 0 | 4 (20.0) | 1 (5.0) | 2 (50.0) | 0 | 1 (25.0) | 0 | 10 (17.5) | 1 (1.8) |
| Blood creatinine increased | 0 | 0 | 0 | 0 | 0 | 0 | 1 (20.0) | 0 | 1 (33.3) | 0 | 1 (20.0) | 0 | 2 (22.2) | 0 | 2 (10.0) | 0 | 1 (25.0) | 0 | 0 | 0 | 8 (14.0) | 0 |
| Blood bilirubin increased | 0 | 0 | 1 (33.3) | 0 | 0 | 0 | 0 | 0 | 0 | 0 | 0 | 0 | 2 (22.2) | 0 | 3 (15.0) | 0 | 2 (50.0) | 1 (25.0) | 0 | 0 | 8 (14.0) | 1 (1.8) |
| Hyponatremia | 0 | 0 | 0 | 0 | 0 | 0 | 0 | 0 | 0 | 0 | 1 (20.0) | 0 | 1 (11.1) | 0 | 2 (10.0) | 0 | 0 | 0 | 3 (75.0) | 1 (25.0) | 7 (12.3) | 1 (1.8) |
| Pain in extremity | 0 | 0 | 1 (33.3) | 1 (33.3) | 0 | 0 | 1 (20.0) | 1 (20.0) | 0 | 0 | 0 | 0 | 2 (22.2) | 0 | 2 (10.0) | 0 | 1 (25.0) | 0 | 0 | 0 | 7 (12.3) | 2 (3.5) |
| Abdominal distension | 0 | 0 | 1 (33.3) | 0 | 0 | 0 | 1 (20.0) | 0 | 0 | 0 | 2 (40.0) | 0 | 3 (33.3) | 0 | 0 | 0 | 0 | 0 | 0 | 0 | 7 (12.3) | 0 |
| Hypokalemia | 1 (100) | 0 | 0 | 0 | 0 | 0 | 1 (20.0) | 0 | 0 | 0 | 1 (20.0) | 0 | 3 (33.3) | 0 | 0 | 0 | 0 | 0 | 0 | 0 | 6 (10.5) | 0 |
| Protein in urine | 0 | 0 | 0 | 0 | 1 (33.3) | 0 | 1 (20.0) | 0 | 0 | 0 | 1 (20.0) | 0 | 0 | 0 | 2 (10.0) | 0 | 1 (25.0) | 0 | 0 | 0 | 6 (10.5) | 0 |

^a^Includes anemia and hemoglobin decreased.

ALT, alanine aminotransferase; AST, aspartate aminotransferase; BID, twice daily; Gr≥3, grade ≥3; QD, once daily; TRAE, treatment (senaparib)-related adverse event; WBC, white blood cell.

Table S6. Pharmacokinetic parameters by dose group for the dose-escalation, single-dose phase, day 1, cycle 1 (PK analysis set, *N* = 57)

| Senaparib dose group | Senaparib PK parameters: Single-dose phase | | | | | | | |
| --- | --- | --- | --- | --- | --- | --- | --- | --- |
|  | **AUC_0-inf_** (h*ng/mL) | **AUC_0-t_** (h*ng/mL) | **C_max_** (ng/mL) | **T_max_** (h) | **CL/F** (L/h) | **V_d_/F** (L) | **t_½_** (h) | **λ_z_** (1/h) |
| 2 mg QD  (*n/N* = 1/1) |  |  |  |  |  |  |  |  |
| Mean±SD or median (range) | 1754.1 | 1462.4 | 177.0 | 1.0 (1.0-1.0) | 1.1 | 21.3 | 13.0 (13.0-13.0) | 5.3 |
| CV% | – | – | – | – | – | – | – | – |
| 5 mg QD  (*n/N* = 3/3) |  |  |  |  |  |  |  |  |
| Mean±SD or median (range) | 4107.2±2303.2 | 3912.7±2180.7 | 378.7±76.1 | 1.9 (1.9-2.0) | 1.5±0.7 | 15.5±3.5 | 7.7 (5.8-10.8) | 5.2±4.6 |
| CV% | 56.1 | 55.7 | 20.1 | – | 48.2 | 22.5 | – | 88.0 |
| 10 mg QD  (*n/N* = 3/3) |  |  |  |  |  |  |  |  |
| Mean±SD or median (range) | 9954.7±1274.9 | 9053.7±887.5 | 606.7±93.5 | 4.0 (2.0-4.0) | 1.0±0.1 | 19.6±0.8 | 14.4 (11.0-15.2) | 5.2±0.9 |
| CV% | 12.8 | 9.8 | 15.4 | – | 13.8 | 4.0 | – | 17.8 |
| 20 mg QD  (*n/N* = 3/5) |  |  |  |  |  |  |  |  |
| Mean±SD or median (range) | 18 955.2±3835.6 | 16 945.0±11 287.2 | 1453.3±408.1 | 1.9 (1.8-1.9) | 1.7±1.4 | 19.1±4.5 | 11.2 (5.1-17.1) | 3.5±3.1 |
| CV% | 73.0 | 66.6 | 28.1 | – | 83.5 | 23.6 | – | 88.6 |
| 40 mg QD  (*n/N* = 3/3) |  |  |  |  |  |  |  |  |
| Mean±SD or median (range) | 16 904.8±4182.4 | 16 428.2±3762.6 | 1680.0±148.0 | 1.8 (1.0-1.8) | 2.5±0.7 | 30.9±2.6 | 9.3 (7.3-10.2) | 7.9±1.4 |
| CV% | 24.7 | 22.9 | 8.8 | – | 26.4 | 8.5 | – | 18.3 |
| 60 mg QD  (*n/N* = 3/5) |  |  |  |  |  |  |  |  |
| Mean±SD or median (range) | 25 575.8±10 554.7 | 24 929.7±9991.9 | 2476.7±342.7 | 2.0 (2.0-3.6) | 2.6±0.9 | 32.0±8.8 | 8.5 (7.9-9.9) | 8.0±0.9 |
| CV% | 41.3 | 40.1 | 13.8 | – | 34.3 | 27.5 | – | 11.0 |
| 80 mg QD  (*n/N* = 3/9) |  |  |  |  |  |  |  |  |
| Mean±SD or median (range) | 76 255.9±33 943.0 | 62 385.0±22 693.1 | 3860.0±1475.0 | 2.0 (2.0-2.0) | 1.2±0.6 | 30.6±14.5 | 17.0 (13.3-23.7) | 4.1±1.1 |
| CV% | 44.5 | 36.4 | 38.2 | – | 50.6 | 47.5 | – | 27.9 |
| 100 mg QD  (*n/N* = 6/20) |  |  |  |  |  |  |  |  |
| Mean±SD or median (range) | 40 487.6±20 226.7 | 38 505.2±18 191.9 | 3425.0±1516.1 | 2.0 (1.0-4.0) | 3.1±1.6 | 39.6±1.1 | 10.4 (6.6-12.9) | 5.7±3.3 |
| CV% | 50.0 | 47.2 | 44.3 | – | 52.5 | 28.1 | – | 56.9 |
| 120 mg QD  (*n/N* = 4/4) |  |  |  |  |  |  |  |  |
| Mean±SD or median (range) | 68 650.9±89 085.5 | 55 962.4±66 684.5 | 3348.3±2828.8 | 1.9 (1.8-6.0) | 6.3±5.8 | 88.7±86.2 | 11.3 (6.3-23.8) | 3.8±2.9 |
| CV% | 129.8 | 119.2 | 84.5 | – | 92.2 | 97.1 | – | 76.1 |
| 50 mg BID  (*n/N* = 4/4) |  |  |  |  |  |  |  |  |
| Mean±SD or median (range) | 35 588.9±27 788.9 | 12 067.8±5895.4 | 1560.0±564.3 | 0.7 (0.5-3.5) | 2.4±2.0 | 36.4±8.6 | 16.5 (5.7-24.3) | 3.3±2.9 |
| CV% | 78.1 | 48.9 | 36.2 | – | 81.5 | 23.7 | – | 87.9 |

λ_z_, terminal elimination rate constant; AUC_0–inf_, area under the time-concentration curve between time 0 and infinity; AUC_0–t_, area under the time-concentration curve between time 0 and the last measurable concentration; BID, twice daily; CL/F, apparent clearance; C_max_, maximum plasma concentration; CV%, coefficient of variation; *n/N*, number of patients with evaluable data/total number of patients in the cohort; PK, pharmacokinetic; QD, once daily; SD, standard deviation; t_½_, terminal half-life; T_max_, time to maximum plasma concentration; V_d_/F, apparent volume of distribution.

Table S7. Pharmacokinetic parameters by dose group for the dose-escalation continuous dosing and dose-expansion phase,
cycle 1 day 15 (PK analysis set, *N* = 57)

| Senaparib dose group | Senaparib PK parameters: Continuous-dosing and dose-expansion phase | | | | | | | |  |
| --- | --- | --- | --- | --- | --- | --- | --- | --- | --- |
|  | **AUC_0-inf_** (h*ng/mL) | **AUC_0-t_** (h*ng/mL) | **C_max_** (ng/mL) | **T_max_** (h) | **CL_ss_/F** (L/h) | **V_ss_/F** (L) | **t_½_** (h) | **λ_z_** (1/h) | **R_ac_** |
| 2 mg QD (*n/N* = 1/1) |  |  |  |  |  |  |  |  |  |
| Mean±SD or median (range) | 4361.8 | 2753.5 | 203.0 | 2.0 (2.0-2.0) | 0.7 | 16.6 | 16.0 (16.0-16.0) | 4.3 | 1.5 |
| CV% | – | – | – | – | – | – | – | – | – |
| 5 mg QD (*n/N* = 3/3) |  |  |  |  |  |  |  |  |  |
| Mean±SD or median (range) | 9655.4±11 022.2 | 6013.7±5649.0 | 470.7±354.0 | 2.0 (2.0-2.0) | 1.6±1.3 | 19.8±7.7 | 10.3 (6.4-21.4) | 3.4±3.3 | 1.4±0.4 |
| CV% | 114.2 | 93.9 | 75.2 | – | 84.9 | 39.0 | – | 98.4 | 28.9 |
| 10 mg QD (*n/N* = 3/3) |  |  |  |  |  |  |  |  |  |
| Mean±SD or median (range) | 10 553.4±2657.5 | 7506.4±892.6 | 598.7±67.9 | 4.0 (2.0-4.0) | 1.3±0.2 | 24.9±4.3 | 13.2 (9.4-16.8) | 5.6±1.6 | 1.4±0.2 |
| CV% | 25.2 | 11.9 | 11.3 | – | 12.4 | 17.1 | – | 29.5 | 13.8 |
| 20 mg QD (*n/N* = 5/5) |  |  |  |  |  |  |  |  |  |
| Mean±SD or median (range) | 22 122.4±18 526.0 | 16 213.5±9863.9 | 1483.2±487.8 | 2.0 (1.8-2.0) | 1.6±0.8 | 18.1±2.5 | 9.4 (5.5-17.5) | 3.8±3.6 | 1.2±0.2 |
| CV% | 83.7 | 60.8 | 32.9 | – | 51.3 | 13.8 | – | 95.5 | 18.7 |
| 40 mg QD (*n/N* = 3/3) |  |  |  |  |  |  |  |  |  |
| Mean±SD or median (range) | 22 437.1±5342.9 | 19 566.5±3285.1 | 2013.3±280.2 | 1.9 (1.8-2.0) | 2.1±0.4 | 21.3±1.7 | 7.5 (5.5-9.2) | 5.6±4.8 | 1.1±0.1 |
| CV% | 23.8 | 16.8 | 13.9 | – | 19.7 | 7.8 | – | 85.9 | 6.4 |
| 60 mg QD (*n/N* = 5/5) |  |  |  |  |  |  |  |  |  |
| Mean±SD or median (range) | 19 189.5±10 074.3 | 17 777.2±8739.1 | 2056.0±975.0 | 2.0 (1.9-3.9) | 4.6±3.6 | 32.1±13.0 | 5.8 (3.4-7.7) | 1.9±3.9 | 1.1±0.0 |
| CV% | 52.5 | 49.2 | 47.4 | – | 77.4 | 40.5 | – | 206.8 | 4.6 |
| 80 mg QD (*n/N* = 9/9) |  |  |  |  |  |  |  |  |  |
| Mean±SD or median (range) | 48 747.0±33 189.7 | 37 727.0±21 476.1 | 3487.8±1649.2 | 2.0 (1.9-3.8) | 2.9±1.8 | 38.8±23.5 | 9.7 (5.8-14.4) | 5.1±3.1 | 1.2±0.1 |
| CV% | 68.1 | 56.9 | 47.3 | – | 62.3 | 60.5 | – | 60.0 | 11.3 |
| 100 mg QD (*n/N* = 20/20) |  |  |  |  |  |  |  |  |  |
| Mean±SD or median (range) | 40 080.4±16 731.2 | 33 827.1±12 273.6 | 3212.5±765.1 | 1.9 (1.8-4.1) | 3.5±1.8 | 40.1±21.9 | 7.5 (5.3-16.1) | 4.5±3.9 | 1.2±0.1 |
| CV% | 41.7 | 36.3 | 23.8 | – | 52.6 | 54.6 | – | 86.5 | 11.0 |
| 120 mg QD (*n/N* = 4/4) |  |  |  |  |  |  |  |  |  |
| Mean±SD or median (range) | 87 799.1±11 6874.0 | 62 737.2±74517.1 | 5107.5±4893.2 | 2.0 (1.9-2.1) | 4.3±3.3 | 1.3±39.5 | 8.8 (6.0-15.2) | 5.1±3.7 | 1.2±0.2 |
| CV% | 133.1 | 118.8 | 95.8 | – | 75.6 | 77.0 | – | 72.0 | 15.4 |
| 50 mg BID (*n/N* = 4/4) |  |  |  |  |  |  |  |  |  |
| Mean±SD or median (range) | 38 418.9±19 776.1 | 17 959.6±5287.0 | 2152.5±354.3 | 2.0 (1.9-3.7) | 2.8±0.8 | 39.9±3.4 | 9.7 (7.8-15.2) | 7.1±2.2 | 1.8±0.4 |
| CV% | 51.5 | 29.4 | 16.5 | – | 29.3 | 8.6 | – | 31.0 | 22.3 |

λ_z_, terminal elimination rate constant; AUC_0-inf_, area under the time-concentration curve between time 0 and infinity; AUC_0-t_, area under the time-concentration curve between time 0 and the last measurable concentration; BID, twice daily; CL_ss_/F, apparent clearance at steady state; C_max_, maximum plasma concentration; CV%, coefficient of variation; *n/N*, number of patients with evaluable data/total number patients in the cohort; PK, pharmacokinetic; QD, once daily; R_ac_, accumulation ratio; SD, standard deviation; t_½_, terminal half-life; T_max_, time to maximum plasma concentration; V_ss_/F, apparent volume of distribution at steady state.

## **Table S8.** Tumor response to senaparib and evaluation of clinical benefit (dose-escalation and dose-expansion periods) in the subgroup of patients in the ITT population with a target lesion at baseline and at least one imaging examination and BRCA^mut+^ after treatment (*n* = 26 evaluable for response).

| Efficacy indicators for BRCA^mut+^ population | Dose groups | | | | | | | | | | Total *N* = 57 |
| --- | --- | --- | --- | --- | --- | --- | --- | --- | --- | --- | --- |
|  | **2 mg QD *n* = 1** | **5 mg QD  *n* = 3** | **10 mg QD *n* = 3** | **20 mg QD  *n* = 5** | **40 mg QD  *n* = 3** | **60 mg QD  *n* = 5** | **80 mg QD  *n* = 9** | **100 mg QD  *n* = 20** | **120 mg QD  *n* = 4** | **50 mg BID  *n* = 4** |  |
| Evaluable, *n* | 0 | 0 | 1 | 2 | 0 | 4 | 6 | 11 | 2 | 0 | 26 |
| ORR,^a^ *n* (%) | – | – | 0 (0) | 1 (50.0) | – | 1 (25.0) | 3 (50.0) | 2 (18.2) | 0 (0) | – | 7 (26.9) |
| (95% CI) | – | – | – | (1.3-98.7) | – | (0.6-80.6) | (11.8-88.2) | (2.3-51.8) | – | – | (11.6-47.8) |
| Complete response | – | – | 0 | 0 | – | 0 | 0 | 0 | 0 | – | 0 |
| Partial response | – | – | 0 | 1 (50.0) | – | 1 (25.0) | 3 (50.0) | 2 (18.2) | 0 | – | 7 (26.9) |
| Stable disease^b^ | – | – | 0 | 0 | – | 3 (75.0) | 2 (33.3) | 6 (54.5) | 1 (50.0) | – | 12 (46.2) |
| DCR,^c^ *n* (%) | – | – | 0 | 1 (50.0) | – | 4 (100) | 5 (83.3) | 8 (72.7) | 1 (50.0) | – | 19 (73.1) |
| (95% CI) | – | – | – | (1.3-98.7) | – | (39.8-100) | (35.9-99.6) | (39.0-94.0) | (1.3-98.7) | – | (52.2-88.4) |
| DOR, days |  |  |  |  |  |  |  |  |  |  |  |
| Evaluable, *n* | 0 | 0 | 0 | 1 | 0 | 1 | 3 | 2 | 0 | 0 | 7 |
| Events, *n* (%) | 0 | 0 | 0 | 0 | 0 | 0 | 3 (100) | 1 (50.0) | 0 | 0 | 4 (57.1) |
| Median (95% CI)^d^ | – | – | – | – | – | – | 120 (39-169) | – (169-NR) | – | – | 169 (39-NR) |
| PFS, days |  |  |  |  |  |  |  |  |  |  |  |
| Evaluable, *n* | 0 | 2 | 1 | 3 | 0 | 4 | 6 | 13 | 2 | 1 | 32 |
| Events, *n* (%) | 0 (0) | 2 (100) | 1 (100) | 1 (33.3) | – | 2 (50.0) | 5 (83.3) | 5 (38.5) | 1 (50.0) | 0 (0) | 17 (53.1) |
| Median (95% CI)^e^ | – | 254.5 (45-464) | 49 (49-49) | – (39-NR) | – | 255 (127-NR) | 167 (40-216) | 289 (42-NR) | – (48-NR) | – | 215 (79-464) |

^a^ORR=complete response+partial response.
^b^A best overall response of stable disease was confirmed only if the tumor imaging date of the visit at which stable disease was recorded was ≥42 days from cycle 1 day 1 (first dose).
^c^DCR=complete response+partial response+stable disease.
^d^95% CI calculated using the accurate probability method.
^e^95% CI calculated using the Kaplan-Meier method.

## BID, twice daily; BRCA^mut+^, tumor harboring *BRCA1* and/or *BRCA2* mutations; CI, confidence interval; DCR, disease control rate; DOR, duration of response; ITT, intent to treat; NR, not reached; ORR, objective response rate; PFS, progression-free survival; QD, once daily.

# Supplementary References

1. National Cancer Institute. Common Terminology Criteria for Adverse Events (CTCAE) version 4.0 (4.03). United States Department of Health and Human Services, National Institutes of Health, National Cancer Institute; 2009.
